# Interrogating the immune landscape of microsatellite stable RAS‐mutated colon cancer

**DOI:** 10.1002/1878-0261.70225

**Published:** 2026-02-24

**Authors:** Rodrigo Dienstmann, Eduardo García‐Galea, Alice O'Farrell, Zak Kinsella, Maxime Meylan, Florent Petitprez, Ingrid Arijs, Tom Venken, Hari Ps, Adrian Lärkeryd, Ian Miller, Janick Selves, Nadja Meindl‐Beinker, Fiorella Ruiz‐Pace, Elena Élez, Raquel Comas‐Navarro, Frank Lincoln, Dirk Fey, Gift Nyamundanda, Aoife Nolan, Joern Lewin, Raquel Perez‐Lopez, Jonathan Briody, Kathleen Bennett, Walter Kolch, David Matallanas, Alexander Kel, Enrique Arenas, Joaquín Arribas, Bart Ghesquière, Josep Tabernero, Julie Meilleroux, Deborah McNamara, Ray McDermott, Marvin Lim, Mary O'Reilly, Brian Bird, Lisa Stack, Lucia Moloney, Patrick Morris, Keith Egan, Maciej Milewski, Lars Scheuer, Joachim Behringer, Georg Bolz, Ramon Salazar, Cristina Santos, Andrea Ruiz, Orla Casey, Verena Murphy, Matthias Ebert, Livio Trusolino, Diether Lambrechts, Anguraj Sadanandam, Catherine Sautès‐Fridman, Jochen Prehn, Paolo Nuciforo, Jacques Fieschi, Florence Monville, Darran O'Connor, Wolf Fridman, Annette Byrne

**Affiliations:** ^1^ Oncology Data Science (ODysSey) Group Vall d'Hebron Institute of Oncology (VHIO) Barcelona Spain; ^2^ University of Vic‐Central University of Catalonia Spain; ^3^ Department of Physiology and Medical Physics RCSI University of Medicine and Health Sciences Dublin Ireland; ^4^ School of Pharmacy and Biomolecular Sciences RCSI University of Medicine and Health Sciences Dublin Ireland; ^5^ Centre de Recherche des Cordeliers, INSERM 1138 Sorbonne Université, Université Paris Cité France; ^6^ VIB Centre for Cancer Biology Leuven Belgium; ^7^ Laboratory for Translational Genetics, Department of Human Genetics Leuven Belgium; ^8^ Division of Cancer Biology The Institute of Cancer Research London UK; ^9^ Data Science & AI, The Centre for Immunotherapy of Cancer, Division of Radiotherapy and Imaging, The Institute of Cancer Research London UK; ^10^ Department of Pathology Institut Universitaire du Cancer‐Oncopole Toulouse France; ^11^ Université Toulouse III‐Paul Sabatier France; ^12^ Department of Medicine II, University Medical Center Mannheim, Medical Faculty Mannheim Heidelberg University Germany; ^13^ Medical Oncology Department, Vall d'Hebron University Hospital and Institute of Oncology (VHIO) Universitat Autònoma de Barcelona Spain; ^14^ Systems Biology Ireland, School of Medicine University College Dublin Ireland; ^15^ Epigenomics AG Berlin Germany; ^16^ Radiomics Group, Vall d'Hebron Institute of Oncology (VHIO) Spain; ^17^ Data Science Centre, School of Population Health RCSI University of Medicine and Health Sciences Dublin Ireland; ^18^ Conway Institute of Biomolecular and Biomedical Research University College Dublin Belfield Ireland; ^19^ geneXplain GmbH Wolfenbüttel Germany; ^20^ Preclinical Research Program Vall d'Hebron Institute of Oncology (VHIO) Spain; ^21^ Metabolomics Expertise Center Leuven Belgium; ^22^ Gastrointestinal and Endocrine Tumor Unit Vall d'Hebron Institute of Oncology (VHIO) Spain; ^23^ Department of Colorectal Surgery, Beaumont Hospital Dublin Ireland; ^24^ Department of Surgery, Royal College of Surgeons in Ireland Dublin Ireland; ^25^ Cancer Trials Ireland Dublin Ireland; ^26^ Department of Medical Oncology Tallaght University Hospital Dublin Ireland; ^27^ Department of Medical Oncology St. Vincent's University Hospital Dublin Ireland; ^28^ Bon Secours Cork Cancer Centre Bon Secours Hospital Cork Ireland; ^29^ Beaumont RCSI Cancer Centre Beaumont Hospital Dublin Ireland; ^30^ Cancer Clinical Trials and Research Unit Beaumont RCSI Cancer Centre Dublin Ireland; ^31^ Onkologische Schwerpunktpraxis Speyer Germany; ^32^ Medical Oncology Department Institut Català d'Oncologia Spain; ^33^ Oncobell Program (IDIBELL) Universitat de Barcelona (Campus Bellvitge) Spain; ^34^ DKFZ‐Hector Cancer Institute at the University Medical Center Mannheim Germany; ^35^ Molecular Medicine Partnership Unit European Molecular Biology Laboratory Heidelberg Germany; ^36^ Candiolo Cancer Institute, FPO IRCCS Torino Italy; ^37^ Department of Oncology University of Torino Italy; ^38^ Molecular Oncology Group Vall d'Hebron University Hospital and Institute of Oncology (VHIO) Barcelona Spain; ^39^ Veracyte SAS Marseille France; ^40^ Present address: Dana‐Farber Cancer Institute Boston Massachusetts USA; ^41^ Present address: Centre for Reproductive Health, Institute for Regeneration and Repair, College of Medicine and Veterinary Medicine The University of Edinburgh UK; ^42^ Present address: Epigenetics Group Josep Carreras Leukaemia Research Institute Barcelona Spain; ^43^ Present address: MImAbs Janvier Group Biosciences Marseille France

**Keywords:** colorectal cancer, immuno‐markers, immunoscore, microsatellite stable, RAS mutation, tumor‐infiltrating lymphocytes

## Abstract

To explore the immune microenvironment of *RAS*‐mutated (*RAS*mt) microsatellite stable (MSS) colon cancer (CC), we retrospectively performed whole exome sequencing, RNA sequencing, and robust digital pathology analyses and studied immune markers in a cohort of 161 patients treated with standard‐of‐care therapies with early stage disease (both fresh frozen and formalin‐fixed paraffin‐embedded [FFPE] samples) or 121 patients with metastatic setting (primary tumor FFPE samples). Only a small proportion of cases exhibited a highly infiltrated immune microenvironment, with a strong association between Immunoscore^®^ (IS)‐high (13% of the samples) and Tumor Lymphocytes Infiltrating Score (TuLIS)‐high scores (25% of the samples). Immunoscore Immune‐Checkpoint (ISIC)‐high tumors (52% of the samples) shared a similar microenvironment composition to IS‐high and TuLIS‐like high tumors and displayed higher mutational burdens than ISIC‐low tumors. In conclusion, a substantial proportion of MSS *RAS*mt CCs exhibit high ISIC scores, meriting evaluation in prospective trials of immunotherapy‐based combination regimens.

## Graphical abstract

COLOSSUS project RAS‐mutated MSS colon cancer study explored transcriptomics and immune cell density by immunohistochemistry (IHC), Immunoscore (IS), ISIC/TuLIS scores, mutation counts, and detected different prevalences but similar microenvironment composition across immune markers with clinical relevance for future immunotherapy combination strategies.

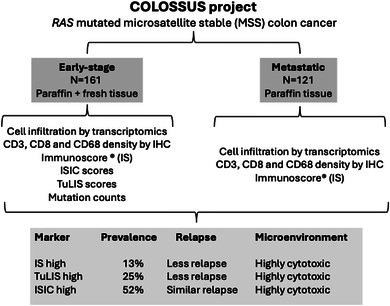

**Abbreviations**
CC: colon cancer
CT: core of tumor
DFS: disease‐free survival
dMMR: deficient mismatch repair
FFPE: formalin‐fixed paraffin‐embedded
IHC: immunohistochemistry
IM: invasive margin
IS: Immunoscore^®^
ISIC: Immunoscore Immune‐Checkpoint
MCP: microenvironment cell populations
MSI: microsatellite instability‐high
MSS: microsatellite stable
OS: overall survival
PFS: progression‐free survival
pMMR: proficient MMR
RASmt: RAS mutated
TMB: tumor mutational burden
TuLIS: Tumor Lymphocytes Infiltrating Score
WES: whole exome sequencing

## 1 Introduction

The efficacy of immunotherapy in colon cancer (CC) has been limited to a subset of patients with microsatellite instability‐high (MSI) or deficient mismatch repair (dMMR) tumors, with response rates close to 70% [1]. However, most CC tumors (85% early stage disease to 95% metastatic disease) are classified as microsatellite stable (MSS) or proficient MMR (pMMR). These tumors respond to immunotherapy in less than 10% of the cases, which has been linked to low tumor mutational burden (TMB), an immunosuppressive microenvironment, and oncogenic pathways that weaken T‐cell activity, particularly in tumors with activating mutations of *KRAS* and other MAPK‐activating alterations [2, 3, 4].

Strategies to improve immunotherapy outcomes in MSS CC include the use of double immune checkpoint blockage with anti‐PD1/−L1 and anti‐CTLA4 agents in the neoadjuvant setting [5] and identifying patient subgroups likely to benefit from combination treatments in the metastatic context. The AtezoTRIBE study showed that adding the PD‐L1 inhibitor atezolizumab to chemotherapy and bevacizumab improved progression‐free survival (PFS) in metastatic (m)CC patients, especially those with high TMB or high Immunoscore Immune‐Checkpoint (ISIC) scores (densities of PD‐L1+ and CD8+ T cells, as well as their proximity in the tumor microenvironment) [6, 7]. The POCHI trial demonstrated promising results with anti‐PD1 pembrolizumab, chemotherapy, and bevacizumab in MSS CC patients with high tumor‐infiltrating lymphocytes [8]. Here, Immunoscore^®^ (IS) (density of CD3+ and CD8+ T cells in the core of tumor [CT] and invasive margin [IM]), and Tumor Lymphocytes Infiltrating Score (TuLIS) (based on CD3 density in the IM) in primary tumor resection specimens were used for patient selection to enrich for tumors with a high T‐cell infiltration. Notably, nearly 70% of the patients enrolled in these trials had *RAS*mt mCC [6, 8].

Ultimately, successful immunotherapy drug‐biomarker codevelopment in CC strongly depends on a deeper understanding of the immune landscape of MSS *RAS*mt disease. To address this, the EU‐funded COLOSSUS project identified 282 CC patients with confirmed MSS *RAS*mt disease, not exposed to immunotherapies (Fig. S1).

## 2 Material and methods

Two parallel patient cohorts were utilized for this study; one retrospective with early stage tumors at diagnosis and another ambispective with patients harboring metastatic disease. Table S1 summarizes the patients' clinical and pathological characteristics. Only primary tumor samples were analyzed in this study, including those from patients with metastatic disease. Several tests were conducted in one or both cohorts, as described in detail below. RNA sequencing (RNAseq) in both cohorts for immune cell composition phenotypes (based on microenvironment cell populations MCP counter algorithm), whole exome sequencing (WES) in the retrospective early stage cohort for confirmation of RASmt status and mutational burden, immunohistochemistry (IHC) for CD3 T cell, CD8 T cell, and CD68 macrophage markers in the center of the tumor (CT) and invasive margin (IM) in both cohorts, in addition to Immunoscore (IS) classes and Immunoscore Immune Checkpoint (ISIC) classes based on digital pathology algorithms in the retrospective early stage cohort, were performed. A graphical overview of the COLOSSUS Project data workflow, including where sample analyses took place, is shown in Fig. S1.

### 2.1 Patient cohorts and clinical data

The study included 282 colon cancer (CC) patients with confirmed microsatellite stable (MSS) RASmt CC tumors. MSS and RAS status were determined at the respective local providing institutions using gold standard diagnostic assays. Availability of primary tumor tissue for comprehensive molecular profiling was mandatory in both cohorts, and patients with rectal cancer treated with neoadjuvant therapy before primary tumor resection were excluded. The mean age of participants was 70 (standard deviation sd 12 years) and 66 (sd 10 years) in the retrospective early stage and the ambispective metastatic cohort, respectively.

The retrospective early stage cohort comprised 161 selected cases from different biobanks (the Bowel Disease Bio‐resource, Beaumont Hospital, Dublin, Ireland; Centre De Ressources Biologiques–Cancer, Centre Hospitalier Universitaire De Toulouse, Toulouse, France; and the Vall d'Hebron Institute of Oncology bioresource), with both fresh frozen tissue and formalin‐fixed paraffin‐embedded (FFPE) primary tumor resections available. Fresh frozen samples were profiled with RNAseq for immune cell composition phenotypes (based on MCP counter) and WES for mutational burden, while FFPE samples were used for IHC analysis of CD3, CD8, and CD68 in CT and IM, as well as for IS and ISIC determination using digital pathology algorithms (Veracyte, Marseille, France).

The ambispective metastatic disease cohort included 121 cases: 28 patients with MSS RASmt metastatic CC recruited in a prospective translational clinical study (NCT03699111) with first‐line systemic treatment for advanced disease including fluoropyrimidines and oxaliplatin, with or without bevacizumab, from April 2019 to October 2021, and 93 additional patients fulfilling the same inclusion criteria identified retrospectively from local biobanks. Prospective patients were recruited at multiple European centers (including Vall d'Hebron Institute of Oncology and Institut Català d'Oncologia, Barcelona, Spain; University Hospital Mannheim and Onkologische Schwerpunktpraxis, Speyer, Germany; Beaumont Hospital, Tallaght University Hospital, St Vincent's University Hospital, Dublin, and Bon Secours Hospital, Cork, Ireland), and all had resected primary tumors with FFPE samples available for molecular profiling. The additional 93 metastatic cases were retrieved from the Vall d'Hebron Institute of Oncology bioresource and Institut Català d'Oncologia.

Clinical and pathological data for all patients were curated using a standardized case report form. At each contributing site, trained staff manually extracted data from electronic medical records and transferred them to a study‐specific case report form that was shared with Cancer Trials Ireland for quality monitoring (completeness and conformance). The Vall d'Hebron Institute of Oncology integrated the clinical data and performed additional checks for plausibility and consistency across sites. Collected variables included sex, age, tumor location (right vs left colon), stage at diagnosis, histological type and grade, perineural invasion, exposure to adjuvant or first‐line palliative chemotherapy, and relapse/recurrence status, which were summarized in Table S1.

The study was conducted in accordance with the principles of the Declaration of Helsinki and was approved by the Human Research Ethics Committees at all participating tissue‐providing biobanks and hospitals. Written informed consent was obtained from all participants at the time of tissue collection or study enrolment. A waiver of reconsent was granted for use of samples and data from local tumor biobanks when patients were deceased or not contactable.

### 2.2 Whole‐exome sequencing

Mutational profiling was performed by WES using an in‐house protocol. Whole‐genome DNA libraries were prepared with the KAPA HyperPlus kit (Roche, Cape Town, South Africa), and exome fragments were captured using the SureSelect Human All Exon V7 kit (Agilent, Hangzhou, China). Libraries were sequenced on a NovaSeq (Illumina, San Diego, CA, USA) platform using 2 × 150 bp paired‐end runs aiming for mean coverage greater than 40×.

Sequence data were analyzed using an in‐house bioinformatics pipeline. After quality control, raw reads were mapped to the human reference genome GRCh38 using Burrows‐Wheeler Aligner, followed by duplicate removal with Picard, indel realignment, and base quality recalibration with GATK (version 4.1.2.0). Somatic variants were called using MuTect2 and annotated with Annovar. Because germline samples were not available, we applied stringent filtering based on public databases: variants present in gnomAD with allelic frequency greater than 0.001 were removed, and only variants with coverage of at least 10×, allelic frequency greater than 0.1, and more than two alternative reads were retained. Finally, we selected known variants in Cancer Consensus Genes according to the COSMIC database.

### 2.3 Bulk RNA sequencing and MCP counter

For frozen samples, RNA extraction and library preparation followed an in‐house pipeline, and for FFPE samples RNA was extracted using the Qiagen RNeasy FFPE Extraction kit according to the manufacturer's instructions. RNA pellets were resuspended in RNase‐free water, quantified by spectrophotometry, and quality‐checked using a bioanalyzer. RNA sequencing libraries were generated with the QuantSeq 3′ mRNA‐Seq Library Prep Kit (FWD, Lexogen, Vienna, Austria) according to the manufacturer's protocol. Briefly, poly‐A‐containing mRNA was purified from total RNA (250 ng when available) by oligo‐dT priming (first‐strand synthesis), followed by second‐strand synthesis with random primers containing 5' Illumina‐compatible linker sequences. Double‐stranded cDNA was purified using magnetic beads, amplified by PCR to introduce full adapter sequences, and sequenced on Illumina platforms (HiSeq 4000, 1 × 51 bp; or NovaSeq, 1 × 100 bp).

Transcriptome profiles were generated using an in‐house bioinformatics pipeline. Reads in FASTQ files were mapped to the human reference genome using HISAT2, and gene‐level counts were obtained using featureCounts to create a raw count matrix. Raw counts were normalized using DESeq2 and compared to Trimmed‐mean of M‐values; batch effects arising from multi‐center data were corrected using ComBat [9, 10, 11]. Normalized expression values were then used as input for tumor microenvironment deconvolution with MCP‐counter (version 1.2.0) [12], which computes abundance scores for eight immune populations (T cells, CD8+ T cells, cytotoxic lymphocytes, natural killer cells, B‐cell lineage, monocytic lineage, myeloid dendritic cells and neutrophils) and two stromal populations (endothelial cells and fibroblasts).

For each cohort separately (retrospective and ambispective), *K*‐means clustering (*k* = 3) was performed on MCP‐counter scores to define three tumor microenvironment clusters: ‘Immune Low’ tumors with low scores across immune populations, ‘Stromal’ tumors with moderate immune infiltration and high fibroblast scores, and ‘Immune High’ tumors with high immune and endothelial scores and low fibroblast scores.

### 2.4 Immunoscore (IS), ISIC and TuLIS

For IS, two adjacent 4 μm FFPE sections from each tumor were stained with antibodies against CD3 and CD8 (clones HDX1 and HDX2, Veracyte, Marseille, France). IHC was performed on a Benchmark XT instrument (Ventana, Tucson, AZ, USA) using the Ultraview Universal DAB IHC Detection Kit and counterstaining with Mayer's hematoxylin. Slides were digitized at 10× magnification (0.45 μm per pixel) using a NanoZoomer XR scanner (Hamamatsu, Japan). Digital image analysis (Immunoscore Analyzer, Veracyte) was used for automatic tumor detection, generation of a 360 μm‐wide invasive margin on each side of the tumor–stroma interface, and quantification of CD3+ and CD8+ cell densities in the CT and IM. In line with Immunoscore^®^ requirements and workflow quality control, samples were excluded if FFPE block quality was insufficient, sample identification was uncertain, tissue was torn or folded, staining background was high, CT or IM regions were missing, or staining intensity was too low. CD3+ and CD8+ cell densities were converted into IS percentiles (0–100%), and tumors were categorized into IS Low (mean percentile 0–70%) and IS High (mean percentile > 70–100%). For ISIC, one 4 μm FFPE section per tumor was stained with antibodies against CD8 and PD‐L1 (clones HDX2 and HDX3, Veracyte) on a Benchmark XT instrument after standard deparaffinization, Cell Conditioning 1 for 54 min, one‐hour incubations at 37 °C with anti‐PD‐L1 and anti‐CD8, and counterstaining with Hematoxylin II for 8 min. Anti‐PD‐L1 and anti‐CD8 antibodies were revealed using the OptiView DAB IHC Detection Kit and UltraView Universal Alkaline Phosphatase Red Detection Kit, respectively. Every slide was scanned at 20× with a NanoZoomer XR scanner, and whole‐slide images were analyzed in HALO (Indica Labs, Corrales, NM, USA) for tissue detection, tumor core definition, and quantification of stained cells. Cell coordinates and phenotypes were exported for spatial analysis, including CD8+ and PD‐L1+ cell densities, cell proximity, and clustering using a 20 μm distance threshold. An ISIC score was computed using a LASSO Cox‐based algorithm and dichotomized into ISIC High (low‐risk group with high marker values) and ISIC Low (high‐risk group with low marker values) [7]. Given the absence of a standardized definition for TuLIS‐high tumors, we defined ‘TuLIS‐like high’ tumors based on CD3 density within the IM as measured by IS, using the upper quartile of the CD3 density distribution as the cutoff.

### 2.5 CD68 immunohistochemistry and digital image analysis

Single‐plex CD68 IHC was performed on 4 μm tissue sections cut using a Leica RM2135 microtome. Staining was carried out on a Bond‐III immunostainer (Leica Biosystems, Newcastle, UK) using primary anti‐CD68 (clone KP1, Agilent, M0814) diluted 1:4000 in Bond Primary Antibody Diluent (Leica, AR9352). Antigen retrieval was performed on the Bond‐III using Bond Epitope Retrieval Solution I (Leica, AR9961) for 10 min, and detection was achieved using the Bond Polymer Refine Detection Kit (Leica, DS9800) with Bond DAB Enhancer (Leica, AR9432); tissues were counterstained with hematoxylin and coverslipped.

Stained sections were scanned at 20× (0.5025 μm per pixel) using a Leica Aperio AT2 slide scanner, and images were stored in .svs format. Digital pathology analyses were performed in QuPath (version 0.5.1) [13]. Color deconvolution (Ruifrok and Johnston method) was calibrated on representative staining areas and applied to all images [14]. The invasive area was manually annotated and programmatically expanded by 500 μm to capture the invasive front, with directionality and annotation quality confirmed under pathologist supervision.

Watershed cell detection was applied with tuned parameters (optical density sum; requestedPixelSizeMicrons = 0.5, backgroundRadiusMicrons = 25.0, backgroundByReconstruction = true; sigmaMicrons = 1.5; minAreaMicrons = 6.0; maxAreaMicrons = 400.0; threshold = 0.2; maxBackground = 3.0; cellExpansionMicrons = 3.0; includeNuclei = false), and positive cells were defined using a Cell DAB OD mean threshold of 0.145. Detection settings were batch‐applied to all images. Resulting data were exported and cleaned in RStudio using tidyverse (version 2.0.0).

### 2.6 Statistical analysis

Descriptive statistics were used to summarize clinicopathological and molecular variables. For discrete variables, chi‐square tests were used when all expected cell counts were at least 5, and Fisher's exact test was applied otherwise. For continuous variables, the Kruskal–Wallis test was used for comparisons across more than two groups, followed by pairwise Wilcoxon rank‐sum tests with false discovery rate (FDR) correction, and the Wilcoxon rank‐sum test was used for two‐group comparisons. For graphical illustration, expression levels of each biomarker (IHC and MCP‐counter scores) within each cohort were ranked from 0 (lowest) to 1 (highest) and plotted accordingly. All direct cross‐comparisons between biomarkers (MCP‐counter clusters, immune cell infiltration, mutation burden, IS, TuLIS, and ISIC classes) were performed only in samples where multiple assays were available on the same lesion, and separately for early stage and metastatic cohorts.

The prognostic impact of these immune markers was assessed. Disease‐free survival (DFS) was defined as the time from colon cancer surgery to disease relapse or death from any cause, whichever occurred first. Progression‐free survival (PFS) in the metastatic setting was measured from start of first‐line systemic therapy to radiological or clinical progression or death, and overall survival (OS) from start of first‐line systemic therapy to death, with patients censored at the date of last known follow‐up. Time‐to‐event endpoints were estimated using Kaplan–Meier methods and compared using log‐rank tests, and Cox proportional hazards models were used to estimate hazard ratios (HRs) with 95% confidence intervals. All reported *P* values are two‐sided. Given the exploratory nature of the study, no global multiple testing correction across all analyses and no multivariable survival modeling were performed. Statistical analyses were conducted in R (version 4.4.2).

## 3 Results

**Fig. 1:**
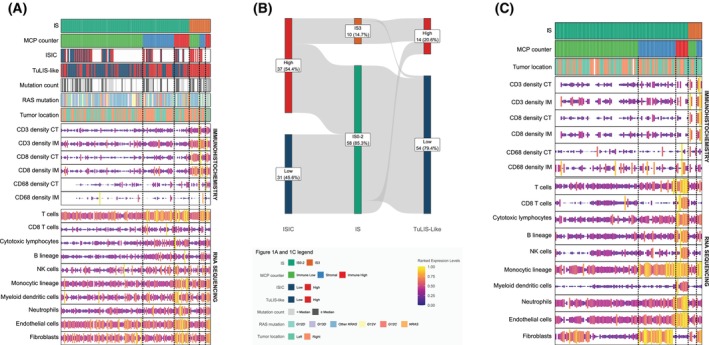
(A) Heatmap with ranked expression levels of different immuno‐markers in 154 samples from the early stage COLOSSUS cohort and associations with genomic or clinical features. IS: Immunoscore; MCP counter: Microenvironment Cell Populations counter signatures; ISIC: Immunoscore Immune‐Checkpoint; TuLIS: Tumor Lymphocytes Infiltrating Score; CT: core of tumor; IM: invasive margin. (B) Alluvial plot with associations between different immuno‐markers in 73 samples from the early stage COLOSSUS cohort. IS: Immunoscore; ISIC: Immunoscore Immune‐Checkpoint; TuLIS: Tumor Lymphocytes Infiltrating Score. (C) Heatmap with ranked expression levels of different immuno‐markers in the 108 samples from the metastatic ambispective COLOSSUS cohort and associations with genomic or clinical features. CT, core of tumor; IM, invasive margin; IS, Immunoscore; ISIC, Immunoscore Immune‐Checkpoint; MCP counter, Microenvironment Cell Populations counter signatures; TuLIS, Tumor Lymphocytes Infiltrating Score.

In the early stage retrospective cohort, the status of IS and MCP‐counter was available in 154 samples. 13% of the cases were IS3 (defined as IS‐high), exhibiting higher CD3/CD8 density in CT and IM (the defining feature of IS3) and higher CD68 density in CT when compared with IS0‐2 tumors (Table S2). Correspondingly, IS3 tumors had higher infiltration scores for cytotoxic lymphocytes and monocytic lineage by MCP counter (Table S3). With regard to MCP‐counter clusters, 60% of the tumors were ‘Immune Low’, 23% were ‘Stromal’ and 13% were ‘Immune‐high’ (Supporting Information). The ‘Immune‐high’ cluster exhibited higher stromal infiltration by cytotoxic lymphocytes, monocytic lineage, natural killer cells, myeloid/dendritic cells, fibroblasts, and endothelial cells (Fig. 1A and Table S4). ‘Immune‐high’ tumors by MCP counter were not enriched in the IS3 subgroup, with 12% of IS0‐2 tumors exhibiting the ‘Immune‐high’ signature (Table 1).

**Table 1:** Association of immunoscore classes and other immuno‐markers.

Characteristic	IS0‐2	IS3	*P*
MCP clusters (Retrospective)			0.2
immune low	81 (64%)	10 (48%)	
Stromal	31 (24%)	6 (29%)	
Immune‐high	15 (12%)	5 (24%)	
Missing	6	0	
ISIC (Retrospective)			0.001
Low	31 (53%)	0 (0%)	
High	27 (47%)	10 (100%)	
Missing	75	11	
TuLIS‐like (Retrospective)			< 0.001
Low	113 (85%)	2 (9.5%)	
High	20 (15%)	19 (90%)	
MCP classes (Ambispective)			0.7
Immune low	60 (63%)	6 (60%)	
Stromal	27 (28%)	4 (40%)	
Immune‐high	9 (9.4%)	0 (0%)	
Missing	2	0	

A subset of 73 tumors (45%) had ISIC scores, with 52% of these classified as ISIC‐high. All samples with IS3 and 47% of those with IS0‐2 were ISIC‐high (Table 1 and Fig. 1A). We found no association between ISIC classes and MCP counter microenvironment clusters (Table S5). ISIC‐high tumors had higher CD3/CD8 density in CT and IM, as well as higher CD68 density in CT when compared with ISIC‐low tumors (Table S6). In addition, ISIC‐high tumors had higher cytotoxic lymphocytes infiltration by MCP‐counter (Table S7).

TuLIS scores were available in 154 samples. As no validated cutoff exists for TuLIS‐high scores, the upper quartile distribution of CD3 density in IM was used to define as TuLIS‐like‐high samples. We found clear enrichment for IS3 in TuLIS‐like‐high tumors, with 90% of IS3 tumors being classified as TuLIS‐like‐high. However, 15% of IS0‐2 were also considered TuLIS‐like‐high (Table 2 and Fig. 1B). TuLIS‐like‐high tumors had higher CD3/CD8 density in both CT and IM, as well as higher CD68 density in CT (Table S8). There were no associations between TuLIS‐like scores and MCP counter clusters (Table S9), although there was higher infiltration of cytotoxic lymphocytes in the microenvironment of TuLIS‐like‐high samples (Table S10). Among 54 TuLIS‐like‐low tumors, 46% were classified as ISIC‐high (Table S11).

**Table 2:** Association of different immuno‐markers with *RAS*mt variant and mutation count in whole exome sequencing.

Characteristic	RAS variant							Mutation count			
	G12D	G12V	G13D	G12C	Other KRAS	NRAS	*P*	Median (absolute)	≤ Median	> Median	*P*
**IS (Retrospective early stage)**							0.3				0.10
IS0‐2	28 (85%)	14 (74%)	17 (94%)	11 (92%)	48 (84%)	14 (100%)		507	39 (91%)	31 (78%)	
IS3	5 (15%)	5 (26%)	1 (5.6%)	1 (8.3%)	9 (16%)	0 (0%)		537	4 (9.3%)	9 (23%)	
Missing	2	1	1	1	1	1			3	2	
**MCP clusters (Retrospective early stage)**							0.7				0.4
Immune low	23 (66%)	11 (55%)	13 (68%)	7 (64%)	36 (64%)	6 (50%)		507	31 (67%)	26 (62%)	
Stromal	8 (23%)	6 (30%)	5 (26%)	4 (36%)	12 (21%)	2 (17%)		526	7 (15%)	11 (26%)	
Immune high	4 (11%)	3 (15%)	1 (5.3%)	0 (0%)	8 (14%)	4 (33%)		467	8 (17%)	5 (12%)	
Missing	0	0	0	2	2	3					
**ISIC (Retrospective early stage)**							0.2				0.074
Low	10 (45%)	3 (27%)	9 (69%)	4 (57%)	7 (39%)	2 (100%)		500	21 (58%)	13 (37%)	
High	12 (55%)	8 (73%)	4 (31%)	3 (43%)	11 (61%)	0 (0%)		523	15 (42%)	22 (63%)	
Missing	13	9	6	6	40	13			10	7	
**TuLIS‐like (Retrospective early stage)**							0.6				0.044
Low	24 (73%)	12 (63%)	15 (83%)	11 (92%)	41 (72%)	11 (79%)		503	37 (86%)	27 (68%)	
High	9 (27%)	7 (37%)	3 (17%)	1 (8.3%)	16 (28%)	3 (21%)		536	6 (14%)	13 (33%)	
Missing	2	1	1	1	1	1			3	2	
**IS (Ambispective metastatic)**							0.5				
IS0‐2	22 (96%)	13 (87%)	9 (100%)	5 (100%)	10 (83%)	4 (80%)					
IS3	1 (4.3%)	2 (13%)	0 (0%)	0 (0%)	2 (17%)	1 (20%)					
Missing	0	1	2	0	2	1					
**MCP clusters (Ambispective metastatic)**							0.14				
Immune low	13 (57%)	10 (63%)	3 (27%)	1 (20%)	9 (64%)	1 (17%)					
Stromal	8 (35%)	6 (38%)	6 (55%)	4 (80%)	5 (36%)	5 (83%)					
Immune High	2 (8.7%)	0 (0%)	2 (18%)	0 (0%)	0 (0%)	0 (0%)					

No associations of immuno‐markers with *RAS*mt variants were found (Table 2). In addition, *RAS*mt variants did not associate with immune cell infiltration by IHC (Table S12) or microenvironment cell composition by MCP‐counter (Table S13). Regarding mutation counts, there was an enrichment for counts above the median in IS3 versus IS0‐2, as well as ISIC‐high and TuLIS‐like‐high versus low (Table 2). Mutation count above the median was associated with higher CD8 T cells in CT and IM (Table S14) but not with MCP‐counter clusters (Table 2) or immune cell composition (Table S15).

In the metastatic ambispective cohort, we analyzed corresponding primary tumor lesions. IS was available for 108 samples, with 9% displaying IS3. Tumors with IS3 had higher density of CD3/CD8 in both CT and IM, higher density of CD68 in CT (Table S16), and higher cytotoxic lymphocytes in MCP counter (Table S17). MCP‐counter clusters were assessed in 119 samples. 61% classified as ‘Immune‐low’, 29% ‘Stromal’, and 10% ‘Immune‐high’. There was no association between IS classes and MCP‐counter clusters (Table 1). As illustrated in Fig. 1C, the subset of ‘Immune‐high’ tumors had higher abundance of cytotoxic lymphocytes, monocytic lineage, B cells, natural killer cells, myeloid and dendritic cells, endothelial cells and fibroblasts (Table S18). We found no association of *RAS*mt variants with IS classes (Table 2), T cell and macrophage IHC infiltration (Table S19), or immune cell composition by MCP‐counter (Table S20). ISIC and TuLIS‐like scores were not available in the metastatic cohort.

**Fig. 2:**
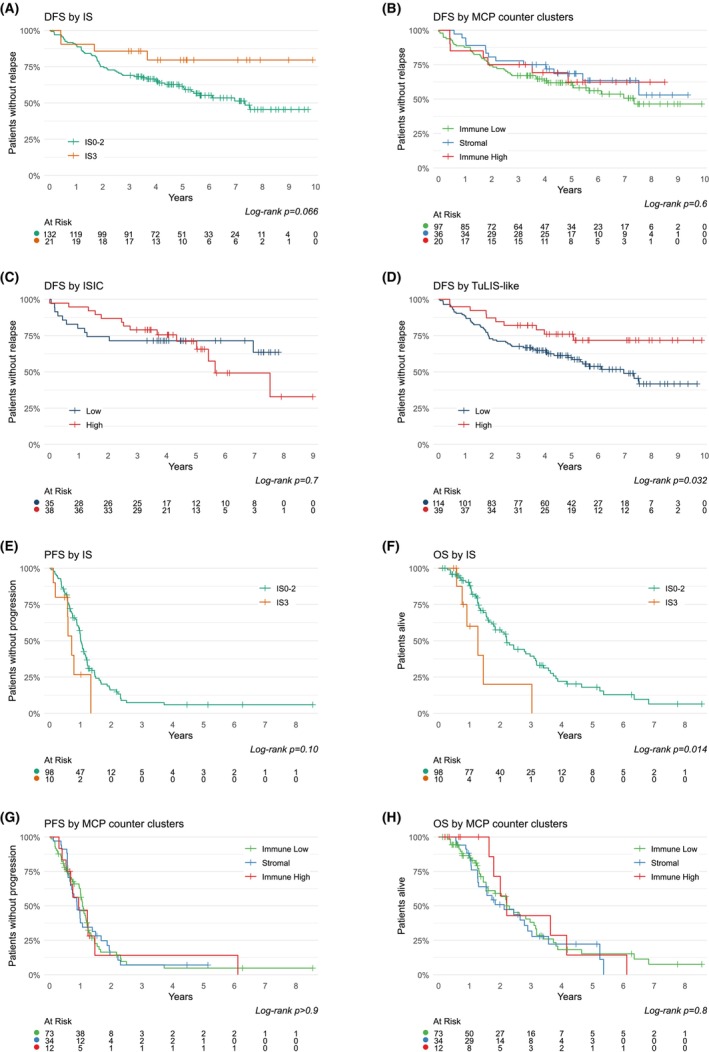
Survival outcomes in the COLOSSUS cohort stratified according to different immuno‐markers (unadjusted Log‐rank test). (A) Disease‐free survival of 153 patients in the early stage colon cancer as per Immunoscore; (B) Disease‐free survival of 153 patients in the early stage colon cancer as per MCP counter clusters. (C) Disease‐free survival of 73 patients in the in early stage colon cancer as per ISIC scores; (D) Disease‐free survival of 153 patients in the early stage colon cancer as per TuLIS‐like scores; (E) Progression‐free survival of 108 patients in the metastatic colon cancer as per Immunoscore; (F) Overall survival of 108 patients in the metastatic colon cancer as per Immunoscore; (G) Progression‐free survival of 119 patients in the in metastatic colon cancer as per MCP counter clusters; (H) Overall survival of 119 patients in the metastatic colon cancer as per MCP counter cluster.

Figure 2 illustrates the outcome associations of different immuno‐markers in MSS *RAS*mt CC. Median follow‐up was 5.5 years in the early stage cohort and 4.2 years in the metastatic population. In the early stage cohort, patients with IS3 tumors had a trend for improved disease‐free survival (DFS) when compared to the IS0‐2 population (Fig. 2A). Likewise, the TuLIS‐like‐high population had favorable DFS when compared to patients with TuLIS‐like‐low tumors (Fig. 2C). Conversely, ISIC score and MCP‐counter clusters had no effect on prognosis (Fig. 2B,D). In the metastatic population, PFS with first‐line oxaliplatin‐based chemotherapy with or without bevacizumab was not affected by IS classes or MCP counter clusters (Fig. 2E). Interestingly, IS3 had a significant detrimental effect on metastatic overall survival (OS) when compared to IS0‐2 groups, an opposite prognostic impact when compared to the favorable DFS association found in the early stage cohort (Fig. 2F). MCP counter clusters had no effect on prognosis in the metastatic cohort (Fig. 2G and Fig. 2H). Liver metastases were diagnosed in 22 out of 43 patients (51%) with systemic relapse in the early stage CC cohort, and in 60 out of 121 (50%) patients with metastatic disease. We did not find associations between the site of metastatic disease and immuno‐markers in either cohort (Table S21).

## 4 Discussion

Our integrated multi‐omics analysis revealed that a small proportion (from 15 to 25%) of MSS *RAS*mt CC tumors exhibited a highly infiltrated immune microenvironment characterized by activated pro‐inflammatory cells, either IS3 or TuLIS‐like‐high. Other studies have already described the immunosuppressive phenotype of MSS and *KRAS* mutated CC, with upregulation of immune checkpoint genes such as CD40, CTLA4, ARG1, STAT3, IDO, and activation of TGFβ signaling, which reduces pro‐inflammatory and cytokine gene signatures [15]. *KRAS* mutated CC typically demonstrate increased neutrophil and M2 macrophage presence, lower infiltration of cytotoxic T cells, helper T cells, regulatory T cells, and B cells compared to their wild‐type counterparts, along with diminished cytolytic and inflammatory responses (2). Furthermore, *KRAS* mutations influence the recruitment and function of immune cells in the tumor microenvironment, with decreased T‐cell infiltration as compared to *KRAS* wild‐type tumors [16, 17].

When compared to IS3 or TuLIS‐like‐high subpopulations of MSS *RAS*mt CC tumors, a larger proportion (52%) were classified as ISIC‐high. Early stage ISIC‐high tumors appeared to share a similar microenvironment composition to IS3 and TuLIS‐like‐high tumors and displayed higher mutation burdens than ISIC‐low counterparts. It is important to emphasize that these associations are based on small numbers from the early stage cohort only. However, if proven predictive of response to immunotherapy combinations in ongoing trials, ISIC scores may potentially expand the population of MSS *RAS*mt tumors eligible for immune checkpoint inhibitors when compared to other biomarkers, such as IS and TuLIS‐high.

Notably, the four immune metrics used (IS, TuLIS, ISIC, and MCP‐counter) capture related but nonidentical aspects of the tumor‐immune microenvironment, so partial discordance between them is expected. Although all four metrics reflect antitumor immunity, they differ in assay platform, cell populations captured, and spatial or transcriptional focus. As a result, they do not fully overlap, and the observed discrepancies likely reflect complementary rather than redundant information on the tumor‐immune microenvironment, thereby enriching the interpretation of our results.

Several limitations should be considered when interpreting these results. First, we lack an MSS *RAS* wild‐type CC cohort for comparative analyses. Direct comparison with KRAS wild‐type tumors was not pursued because *RAS*mt CCs exhibit a distinct, more immunosuppressive tumor microenvironment and prognostic framework, which may confound the interpretation of immune biomarker differences between these groups. In addition, *RAS*mt CC population accounts for about 70% of patients included in trials evaluating chemotherapy–immunotherapy combinations in metastatic colorectal cancer, such as AtezoTRIBE and POCHI [6, 8]. Another drawback is the fact that fresh frozen tissue for RNAseq and WES was only available in the early stage cohort, as were ISIC and TuLIS scores. Different sample materials were used for gene expression profiling in early stage and metastatic populations (fresh frozen and FFPE, respectively), which may affect RNA integrity and the comparability of transcriptomic markers between cohorts. Also, molecular tests were not uniformly applied across the entire cohort, with different subsets of patients subject to distinct molecular and immunological analyses depending on sample availability and quality. This limitation impacts the statistical power for comparisons that involve multiple biomarkers and omics measures. Furthermore, we analyzed only primary tumor samples, despite existing evidence suggesting that *RAS*mt metastatic lesions have a different immune cell composition, with less infiltration of natural killer cells(2). Finally, the study did not have the gold standard TuLIS‐score measurement currently in use to inform clinical trial recruitment, which potentially limits the direct translatability of the findings to those specific trials. Notwithstanding these limitations, the principal strength of this study resides in our comprehensive multi‐omic, immune‐focused analysis of a uniquely characterized cohort of MSS *RAS*mt CC patient samples. The data are of high quality, with robust clinical annotation (including outcomes that align with clinical trial cohorts), digital pathology and molecular profiling performed in reference laboratories. Our findings in the early stage cohort lay the groundwork for investigating immunomodulatory therapies, including (neo)adjuvant treatment strategies, in MSS colorectal cancer. Importantly, our comparative analyses of immune markers are directly applicable to clinical trials evaluating immunotherapy combinations in metastatic settings, either retrospectively or prospectively. Specifically, ongoing and future studies that stratify metastatic MSS CC patients randomized to chemotherapy with or without immune checkpoint inhibitors such as anti‐PD‐1/PD‐L1 and anti‐CTLA‐4 agents will provide decisive insights on which biomarkers best predict therapeutic benefit.

## 5 Conclusion

We observe that the ISIC‐high score identifies a substantially larger subset of tumors as immune‐high compared to IS, and that ISIC‐high tumors share similar immune microenvironment characteristics with IS‐high and TuLIS‐high tumors. Moreover, ISIC‐high tumors are associated with increased tumor mutational burden relative to ISIC‐low tumors, consistent with a biologically relevant immunogenic phenotype. Our findings position ISIC as a promising method for immune stratification in this patient population, pending further clinical validation. Collectively, these findings may inform preclinical research programs and aid in the interpretation of ongoing and future clinical trials involving combinatorial immunotherapeutic strategies, as well as novel targeted therapies tailored to distinct *RAS*mt variants in MSS CC. Ultimately, this work aims to contribute to delivering improved clinical outcomes in this intractable malignancy.

## Conflict of interest

RD declares advisory role for Roche, Foundation Medicine, AstraZeneca, Pfizer, received a speaker's fee from Roche, Foundation Medicine, GuardantHealth, Ipsen, Amgen, Servier, Sanofi, Libbs, Merck Sharp & Dohme, Lilly, AstraZeneca, Johnson and Johnson, Takeda, Bristol Myers Squibb, GlaxoSmithKline, Gilead, Pfizer, research grants from Merck, Novartis, Daiichi‐Sankyo, GlaxoSmithKline, Pfizer and AstraZeneca, and is investor in Trialing Health, S.L. LT has received research grants from Menarini, Merck KGaA, Merus, Pfizer, Servier and Symphogen. JT reports personal financial interest in form of scientific consultancy role for Accent Therapeutics, Alentis Therapeutics, AstraZeneca, Boehringer Ingelheim, Bristol Myers Squibb, Cartography Biosciences, Chugai, Daiichi Sankyo, F. Hoffmann‐La Roche, Genentech, Lilly, Menarini, Merus, MSD, Novartis, Ono Pharma USA, Peptomyc, Pfizer, Pierre Fabre, Quantro Therapeutics, Scandion Oncology, Scorpion Therapeutics, Servier, Sotio Biotech, Taiho, Takeda Oncology and Tolremo Therapeutics. Stocks: Alentis Therapeutics, Oniria Therapeutics, 1TRIALSPand Pangaea Oncology. DL is inventor on a patent related to detecting MSI that was licensed to Biocartis N.V. and meanwhile is FDA‐approved for the treatment of MSI CRC tumors with Opdivo^®^ Plus Yervoy^®^. AS declares Co‐Foundership role in Oncoassign and a Chief Advisor role in Diagnostring Laboratories. He serves on the Advisory Board of Enedra Therapeutics and has a financial interest in Oncoassign and Enedra Therapeutics. WHF declares advisory roles in Anaveon, Catalym, Genenta, IGI ImmunoBiotherpeutics, Mestag, OSE ImmunoPharmaceutical, Oxford Biotherapeutics. JS declares advisory roles at BMS, MSD, AstraZeneca, received a speaker's fee from MSD, BMS and AstraZeneca.

## Author contributions

RD, ACO, and ATB: conceptualization, funding acquisition, data curation, methodology, project administration, supervision, manuscript writing. EGG, FRP: data analysis, visualization. ZK, MM, FP, IA, TV, PH, AL, ISM, JS, NMMB, EE, RCN, FL, DF, GN, AN, JL, RPL, JB, KB, WK, DM, AK, EJA, JA, BG, JT, OC, VM, MPE, LT, DL, AS, CSF, JHP, PN, JF, FM, DPO, WHF, JM, DAM, RM, ML, MO, BB, LS, LM, PGM, KE, MM, LS, JB, GB, RS, CS, and AR: data curation, data analysis, manuscript reviewing and editing.

## Supporting information


**Fig. S1.** COLOSSUS Project Data Workflow.

**Fig. S2.** Illustrative example of CD68 staining.

**Table S1.** Patient and tumor characteristics stratified by COLOSSUS cohort.
**Table S2.** Immunoscore and IHC infiltration markers in the retrospective cohort.
**Table S3.** Immunoscore and MCP counter immune cell composition in the retrospective cohort.
**Table S4.** MCP counter clusters and immune cell composition in the retrospective cohort.
**Table S5.** ISIC classes and MCP counter clusters in the retrospective cohort.
**Table S6.** ISIC classes and IHC infiltration markers in the retrospective cohort.
**Table S7.** ISIC Groups and immune cell composition in the retrospective cohort.
**Table S8.** TuLIS‐like score and IHC infiltration markers in the retrospective cohort.
**Table S9.** TuLIS‐like score and MCP counter clusters in the retrospective cohort.
**Table S10.** TuLIS‐like score and MCP counter immune cell composition in the retrospective cohort.
**Table S11.** TuLIS‐like score and ISIC classes in the retrospective cohort.
**Table S12.** RAS Mutated variant and IHC infiltration markers in the retrospective cohort.
**Table S13.** RAS Mutated variant and MCP counter immune cell composition in the retrospective cohort.
**Table S14.** Mutation count and IHC markers in the retrospective cohort.
**Table S15.** Mutation count and MCP counter immune cell composition in the retrospective cohort.
**Table S16.** Immunoscore and IHC infiltration markers in the ambispective cohort.
**Table S17.** Immunoscore in MCP counter immune cell composition in the ambispective cohort.
**Table S18.** MCP Counter clusters and immune cell composition in the ambispective cohort.
**Table S19.** RAS Mutated variant and IHC infiltration markers in the ambispective cohort.
**Table S20.** RAS Mutated variant and MCP counter immune cell composition in the ambispective cohort.
**Table S21.** Site of metastasis and immuno markers in the retrospective and ambispective cohorts.

## Data Availability

Data are available upon request from the corresponding author.
