# Supplementary figures and images for: Interrogating the immune landscape of microsatellite stable RAS‐mutated colon cancer
Source: Mol Oncol. 2026 Feb 24;20(7):1713–25. doi: 10.1002/1878-0261.70225 (PMC13352957; doi:10.1002/1878-0261.70225)

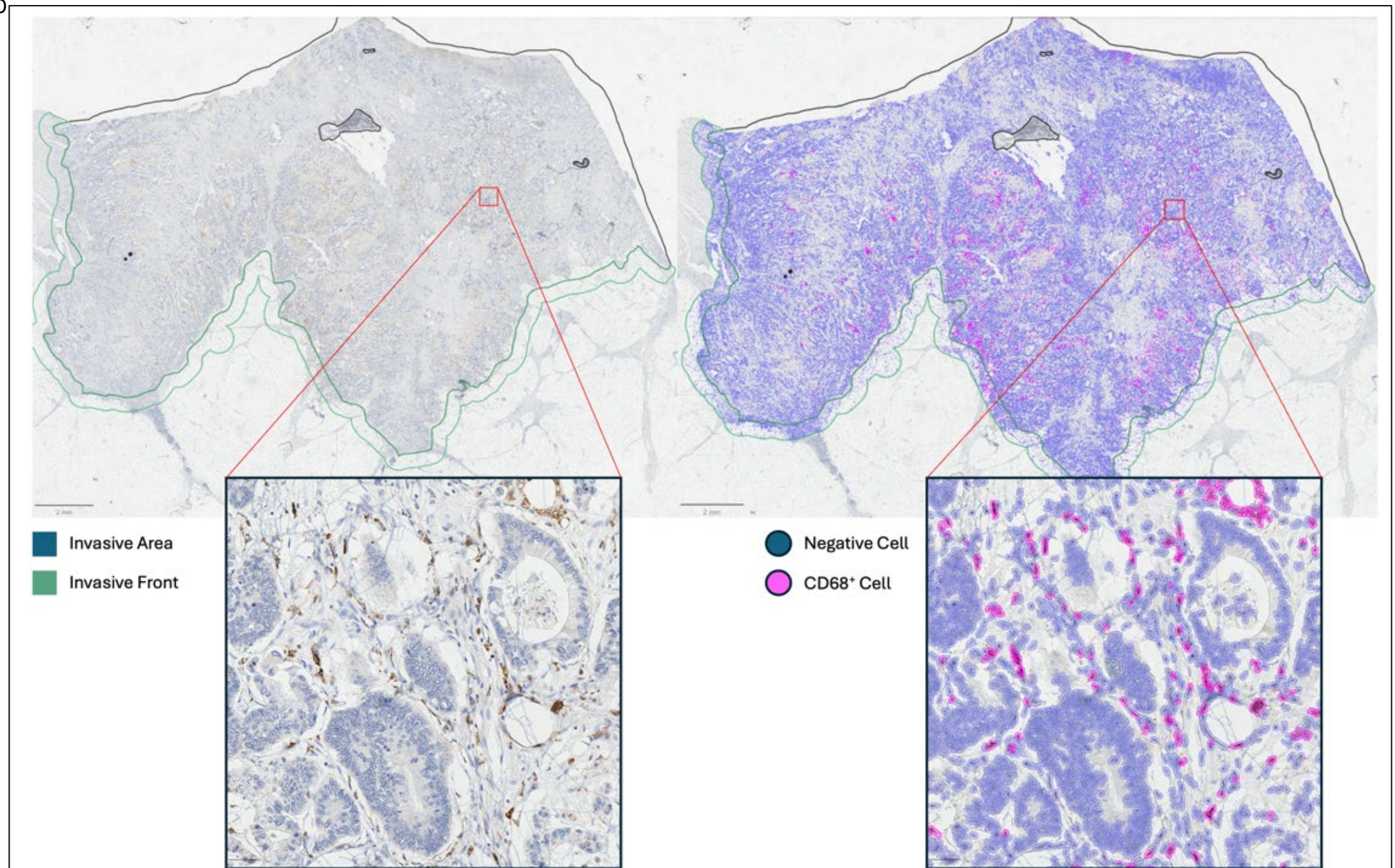

Supplement: Supplementary file 1 — Fig. S1. COLOSSUS Project Data Workflow. [file MOL2-20-1713-s003.pdf]
