# Supplementary material for: Interrogating the immune landscape of microsatellite stable RAS‐mutated colon cancer
Source: Mol Oncol. 2026 Feb 24;20(7):1713–25. doi: 10.1002/1878-0261.70225 (PMC13352957; doi:10.1002/1878-0261.70225)
Supplement: Supplementary file 2 — Fig. S2. Illustrative example of CD68 staining. [file MOL2-20-1713-s001.pdf]

EXTENDED DATA: FIGURE 1

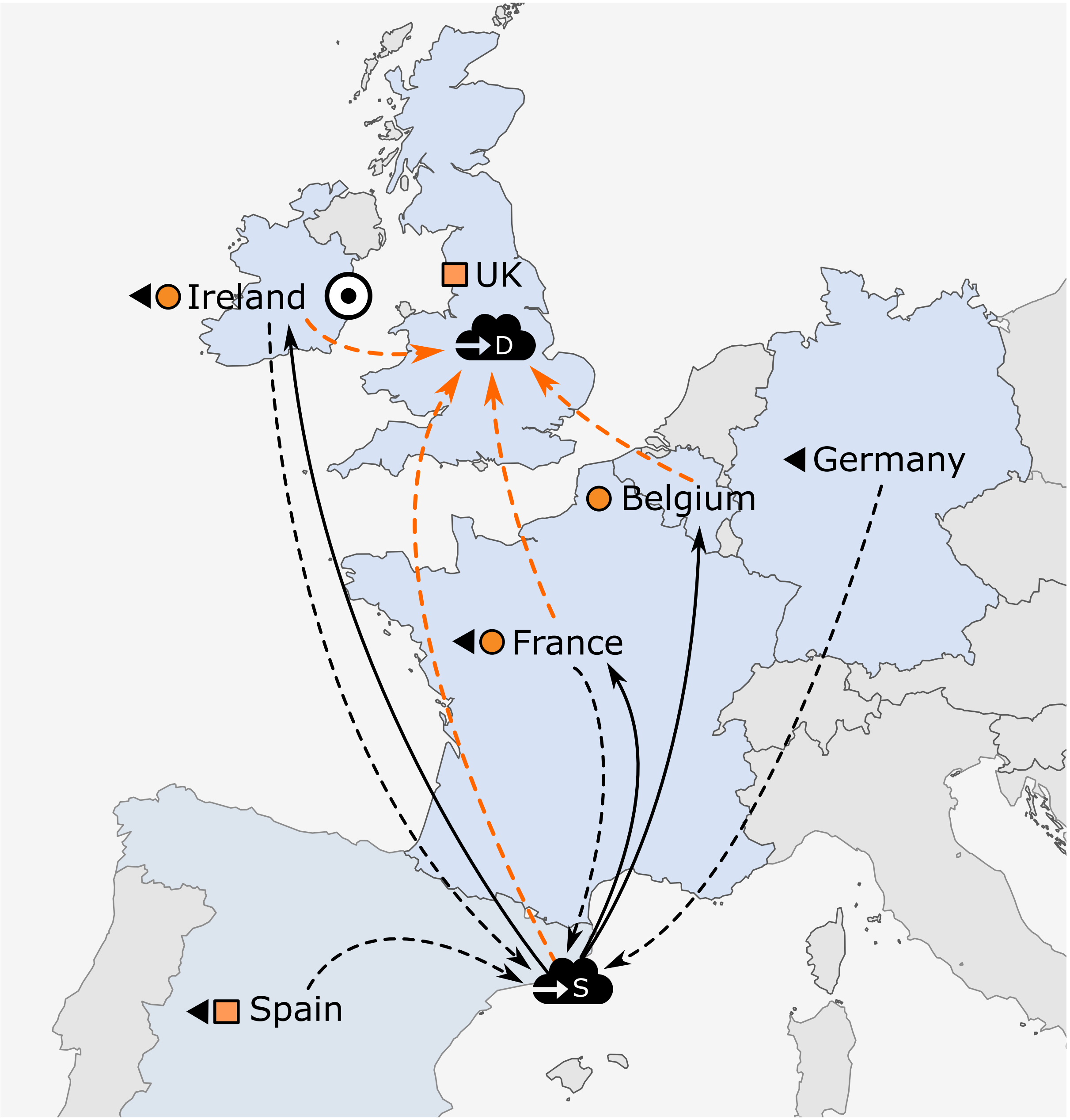

Tissue provinding sites

Germany ✸
France ✸
Spain ✸✸
Ireland ✸✸

✸ Early-stage CC retrospective cohort
✸ Metastatic CC retrospective cohort

Molecular profiling center

VIB (Belgium)	RNA seq / WES
VERACYTE (France)	IS / ISIC / IHC
INSERM (France)	MCP counter
RCSI (Ireland)	IHC

Multi-omics data analysis center

VIHO (Spain)
ICR (UK)

RCSI (Ireland)<br>CTI (Ireland)	Coordinator site<br>Clinical Trial Coordinator
VHIO (Spain)	Sample repository and clinical data management
ICR (UK)	Data repository

Samples submitted to repository
Samples sent for molecular profiling
Data submitted to repository

Workflow

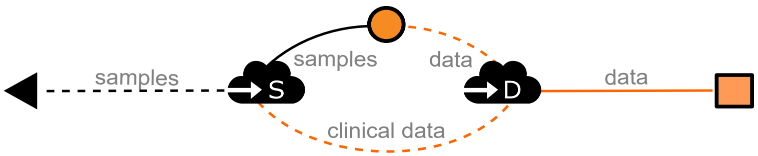

COLOSSUS
