# Supplementary material for: Interrogating the immune landscape of microsatellite stable RAS‐mutated colon cancer
Source: Mol Oncol. 2026 Feb 24;20(7):1713–25. doi: 10.1002/1878-0261.70225 (PMC13352957; doi:10.1002/1878-0261.70225)
Supplement: Supplementary file 3 — Table S1. Patient and tumor characteristics stratified by COLOSSUS cohort. Table S2. Immunoscore and IHC infiltration markers in the retrospective cohort. Table S3. Immunoscore and MCP counter immune cell composition in the retrospective cohort. Table S4. MCP counter clusters and immune cell composition in the retrospective cohort. Table S5. ISIC classes and MCP counter clusters in the retrospective cohort. Table S6. ISIC classes and IHC infiltration markers in the retrospective cohort. Table S7. ISIC Groups and immune cell composition in the retrospective cohort. Table S8. TuLIS‐like score and IHC infiltration markers in the retrospective cohort. Table S9. TuLIS‐like score and MCP counter clusters in the retrospective cohort. Table S10. TuLIS‐like score and MCP counter immune cell composition in the retrospective cohort. Table S11. TuLIS‐like score and ISIC classes in the retrospective cohort. Table S12. RAS Mutated variant and IHC infiltration markers in the retrospective cohort. Table S13. RAS Mutated variant and MCP counter immune cell composition in the retrospective cohort. Table S14. Mutation count and IHC markers in the retrospective cohort. Table S15. Mutation count and MCP counter immune cell composition in the retrospective cohort. Table S16. Immunoscore and IHC infiltration markers in the ambispective cohort. Table S17. Immunoscore in MCP counter immune cell composition in the ambispective cohort. Table S18. MCP Counter clusters and immune cell composition in the ambispective cohort. Table S19. RAS Mutated variant and IHC infiltration markers in the ambispective cohort. Table S20. RAS Mutated variant and MCP counter immune cell composition in the ambispective cohort. Table S21. Site of metastasis and immuno markers in the retrospective and ambispective cohorts. [file MOL2-20-1713-s002.docx]

Supplementary Table 1: Patient and tumor characteristics stratified by COLOSSUS cohort

| **Characteristics** | **Retrospective**  **Early-stage**  **N=161** | **Ambispective**  **Metastatic**  **N=121** |
| --- | --- | --- |
| **Age at diagnosis** |  |  |
| Mean (SD) | 70 (12) | 66 (10) |
| Median (Q1 - Q3) | 73 (62 - 79) | 67 (60 - 73) |
| Min, Max | 26, 91 | 33, 85 |
| **Gender** |  |  |
| Female | 76 (47%) | 52 (43%) |
| Male | 85 (53%) | 69 (57%) |
| **Tumor location** |  |  |
| Right | 86 (53%) | 56 (47%) |
| Left | 75 (47%) | 63 (53%) |
| Missing | 0 | 2 |
| **Stage at diagnosis** |  |  |
| I | 6 (3.7%) | 1 (0.8%) |
| II | 77 (48%) | 18 (15%) |
| III | 78 (48%) | 22 (18%) |
| IV | 0 (0%) | 80 (66%) |
| **Histologic type** |  |  |
| Conventional | 140 (87%) | 98 (81%) |
| Mucinous | 21 (13%) | 22 (18%) |
| Other | 0 (0%) | 1 (0.8%) |
| **Histologic grade** |  |  |
| G1 | 46 (29%) | 25 (22%) |
| G2 | 82 (51%) | 51 (45%) |
| G3 | 33 (20%) | 34 (30%) |
| G4 | 0 (0%) | 3 (2.7%) |
| Missing | 0 | 8 |
| **Adjuvant chemotherapy** |  |  |
| No | 90 (57%) | 81 (83%) |
| Yes | 69 (43%) | 17 (17%) |
| Missing | 2 | 23 |
| **Adjuvant chemotherapy regimen** |  |  |
| XELOX/FOLFOX | 48 (71%) | 2 (25%) |
| 5FU/CPC | 16 (24%) | 6 (75%) |
| Others | 4 (5.9%) | 0 (0%) |
| Missing | 1 | 9 |
| **Treatment regimen first-line** |  |  |
| FOLFOX/XELOX |  | 57 (47%) |
| FOLFOX + Bevacizumab |  | 53 (44%) |
| CPC |  | 8 (6.5%) |
| CPC + Bevacizumab |  | 3 (2.5%) |

Supplementary Table 2: Immunoscore and IHC infiltration markers in the retrospective cohort

| **Characteristic** | **IS0-2** N = 133 | **IS3** N = 21 | **p-value** |
| --- | --- | --- | --- |
| **CD3 Density (CT)** |  |  | <0.001 |
| Mean (SD) | 388 (196) | 926 (342) |  |
| Median (Q1 - Q3) | 359 (246 - 512) | 795 (749 - 1,064) |  |
| Min - Max | 46 - 951 | 600 - 2,127 |  |
| **CD3 Density (IM)** |  |  | <0.001 |
| Mean (SD) | 753 (308) | 1,536 (367) |  |
| Median (Q1 - Q3) | 737 (525 - 925) | 1,457 (1,231 - 1,677) |  |
| Min - Max | 171 - 1,558 | 1,068 - 2,409 |  |
| **CD8 Density (CT)** |  |  | <0.001 |
| Mean (SD) | 116 (80) | 417 (148) |  |
| Median (Q1 - Q3) | 95 (62 - 146) | 364 (329 - 468) |  |
| Min - Max | 16 - 404 | 232 - 735 |  |
| **CD8 Density (IM)** |  |  | <0.001 |
| Mean (SD) | 283 (166) | 744 (204) |  |
| Median (Q1 - Q3) | 253 (159 - 367) | 659 (579 - 910) |  |
| Min - Max | 50 - 886 | 472 - 1,187 |  |
| **CD68 Density (CT)** |  |  | 0.002 |
| Mean (SD) | 189 (189) | 470 (429) |  |
| Median (Q1 - Q3) | 127 (52 - 259) | 395 (146 - 547) |  |
| Min - Max | 3 - 834 | 40 - 1,615 |  |
| Unknown | 18 | 4 |  |
| **CD68 Density (IM)** |  |  | 0.4 |
| Mean (SD) | 215 (410) | 312 (624) |  |
| Median (Q1 - Q3) | 75 (31 - 251) | 123 (50 - 262) |  |
| Min - Max | 1 - 3,368 | 5 - 2,627 |  |
| Unknown | 18 | 4 |  |

Supplementary Table 3: Immunoscore and MCP counter immune cell composition in the retrospective cohort

| **Characteristic** | **IS0-2** N = 133 | **IS3** N = 21 | **p-value** |
| --- | --- | --- | --- |
| **T cells** |  |  | 0.047 |
| Mean (SD) | 1.63 (0.38) | 1.79 (0.30) |  |
| Median (Q1 - Q3) | 1.58 (1.37 - 1.87) | 1.76 (1.56 - 1.97) |  |
| Min - Max | 0.22 - 2.63 | 1.33 - 2.60 |  |
| Unknown | 6 | 0 |  |
| **CD8 T cells** |  |  | 0.6 |
| Mean (SD) | 0.60 (0.72) | 0.61 (0.60) |  |
| Median (Q1 - Q3) | 0.49 (0.03 - 0.95) | 0.63 (0.18 - 0.86) |  |
| Min - Max | -0.41 - 4.18 | -0.34 - 1.99 |  |
| Unknown | 6 | 0 |  |
| **Cytotoxic lymphocytes** |  |  | 0.044 |
| Mean (SD) | 1.63 (0.48) | 1.80 (0.42) |  |
| Median (Q1 - Q3) | 1.59 (1.30 - 1.83) | 1.76 (1.49 - 1.95) |  |
| Min - Max | 0.86 - 3.65 | 1.14 - 2.72 |  |
| Unknown | 6 | 0 |  |
| **B lineage** |  |  | 0.3 |
| Mean (SD) | 1.34 (0.65) | 1.54 (0.73) |  |
| Median (Q1 - Q3) | 1.26 (0.88 - 1.70) | 1.48 (0.89 - 1.93) |  |
| Min - Max | 0.12 - 4.65 | 0.61 - 3.21 |  |
| Unknown | 6 | 0 |  |
| **NK cells** |  |  | 0.3 |
| Mean (SD) | -0.19 (0.44) | -0.07 (0.48) |  |
| Median (Q1 - Q3) | -0.23 (-0.55 - 0.12) | -0.16 (-0.33 - 0.38) |  |
| Min - Max | -0.89 - 1.29 | -0.79 - 0.76 |  |
| Unknown | 6 | 0 |  |
| **Monocytic lineage** |  |  | 0.001 |
| Mean (SD) | 3.22 (0.49) | 3.58 (0.46) |  |
| Median (Q1 - Q3) | 3.20 (2.91 - 3.45) | 3.69 (3.28 - 3.94) |  |
| Min - Max | 1.88 - 5.02 | 2.73 - 4.17 |  |
| Unknown | 6 | 0 |  |
| **Myeloid dendritic cells** |  |  | 0.2 |
| Mean (SD) | 0.09 (0.24) | 0.18 (0.26) |  |
| Median (Q1 - Q3) | 0.07 (-0.10 - 0.23) | 0.12 (-0.01 - 0.38) |  |
| Min - Max | -0.38 - 0.82 | -0.19 - 0.64 |  |
| Unknown | 6 | 0 |  |
| **Neutrophils** |  |  | 0.4 |
| Mean (SD) | 2.06 (0.41) | 2.14 (0.42) |  |
| Median (Q1 - Q3) | 2.02 (1.78 - 2.26) | 2.10 (1.80 - 2.41) |  |
| Min - Max | 1.18 - 3.65 | 1.57 - 3.01 |  |
| Unknown | 6 | 0 |  |
| **Endothelial cells** |  |  | 0.4 |
| Mean (SD) | 2.62 (0.35) | 2.57 (0.33) |  |
| Median (Q1 - Q3) | 2.59 (2.36 - 2.87) | 2.48 (2.36 - 2.72) |  |
| Min - Max | 1.38 - 3.62 | 2.00 - 3.22 |  |
| Unknown | 6 | 0 |  |
| **Fibroblasts** |  |  | 0.7 |
| Mean (SD) | 6.86 (0.97) | 6.95 (0.93) |  |
| Median (Q1 - Q3) | 6.80 (6.04 - 7.51) | 6.86 (6.43 - 7.75) |  |
| Min - Max | 4.19 - 9.32 | 5.44 - 8.32 |  |
| Unknown | 6 | 0 |  |

Supplementary Table 4: MCP counter clusters and immune cell composition in the retrospective cohort

| **Characteristic** | **Immune Low** N = 97 | **Stromal** N = 37 | **Immune High** N = 20 | **p-value** |
| --- | --- | --- | --- | --- |
| **T cells** |  |  |  | <0.001 |
| Mean (SD) | 1.46 (0.27) | 1.94 (0.26) | 2.01 (0.36) |  |
| Median (Q1 - Q3) | 1.48 (1.31 - 1.59) | 1.91 (1.74 - 2.11) | 2.02 (1.76 - 2.25) |  |
| Min - Max | 0.22 - 2.10 | 1.51 - 2.63 | 1.35 - 2.60 |  |
| **CD8 T cells** |  |  |  | <0.001 |
| Mean (SD) | 0.40 (0.54) | 0.96 (0.94) | 0.87 (0.61) |  |
| Median (Q1 - Q3) | 0.22 (-0.01 - 0.69) | 0.86 (0.36 - 1.40) | 0.70 (0.53 - 1.21) |  |
| Min - Max | -0.41 - 2.67 | -0.37 - 4.18 | -0.11 - 2.27 |  |
| **Cytotoxic lymphocytes** |  |  |  | <0.001 |
| Mean (SD) | 1.40 (0.30) | 1.91 (0.34) | 2.26 (0.53) |  |
| Median (Q1 - Q3) | 1.39 (1.15 - 1.63) | 1.83 (1.70 - 2.15) | 2.29 (1.86 - 2.51) |  |
| Min - Max | 0.86 - 2.39 | 1.15 - 2.70 | 1.33 - 3.65 |  |
| **B lineage** |  |  |  | <0.001 |
| Mean (SD) | 1.04 (0.41) | 1.88 (0.61) | 1.86 (0.82) |  |
| Median (Q1 - Q3) | 1.02 (0.73 - 1.30) | 1.72 (1.49 - 2.09) | 1.75 (1.27 - 2.20) |  |
| Min - Max | 0.12 - 2.06 | 0.94 - 3.30 | 0.83 - 4.65 |  |
| **NK cells** |  |  |  | <0.001 |
| Mean (SD) | -0.30 (0.41) | -0.05 (0.42) | 0.17 (0.45) |  |
| Median (Q1 - Q3) | -0.36 (-0.64 - -0.09) | -0.12 (-0.38 - 0.24) | 0.22 (-0.21 - 0.43) |  |
| Min - Max | -0.89 - 1.29 | -0.74 - 0.76 | -0.41 - 1.19 |  |
| **Monocytic lineage** |  |  |  | <0.001 |
| Mean (SD) | 3.11 (0.43) | 3.29 (0.30) | 4.01 (0.44) |  |
| Median (Q1 - Q3) | 3.15 (2.83 - 3.36) | 3.25 (3.08 - 3.50) | 4.05 (3.69 - 4.17) |  |
| Min - Max | 1.88 - 4.28 | 2.67 - 4.17 | 3.28 - 5.02 |  |
| **Myeloid dendritic cells** |  |  |  | <0.001 |
| Mean (SD) | 0.03 (0.21) | 0.14 (0.19) | 0.40 (0.24) |  |
| Median (Q1 - Q3) | -0.01 (-0.11 - 0.15) | 0.11 (0.01 - 0.29) | 0.38 (0.16 - 0.62) |  |
| Min - Max | -0.38 - 0.70 | -0.20 - 0.64 | 0.04 - 0.82 |  |
| **Neutrophils** |  |  |  | <0.001 |
| Mean (SD) | 1.90 (0.28) | 2.19 (0.33) | 2.65 (0.41) |  |
| Median (Q1 - Q3) | 1.88 (1.72 - 2.05) | 2.17 (1.96 - 2.31) | 2.65 (2.30 - 2.90) |  |
| Min - Max | 1.18 - 2.63 | 1.57 - 3.22 | 2.15 - 3.65 |  |
| **Endothelial cells** |  |  |  | <0.001 |
| Mean (SD) | 2.55 (0.32) | 2.53 (0.24) | 3.07 (0.24) |  |
| Median (Q1 - Q3) | 2.50 (2.35 - 2.72) | 2.54 (2.35 - 2.70) | 3.06 (2.88 - 3.18) |  |
| Min - Max | 1.38 - 3.55 | 2.09 - 2.95 | 2.67 - 3.62 |  |
| **Fibroblasts** |  |  |  | <0.001 |
| Mean (SD) | 6.79 (0.93) | 6.48 (0.69) | 8.01 (0.61) |  |
| Median (Q1 - Q3) | 6.72 (6.03 - 7.50) | 6.45 (6.09 - 6.96) | 8.14 (7.68 - 8.35) |  |
| Min - Max | 4.19 - 8.91 | 4.74 - 7.66 | 6.94 - 9.32 |  |

Supplementary Table 5: ISIC classes and MCP counter clusters in the retrospective cohort

|  | **ISIC** | |  |  |
| --- | --- | --- | --- | --- |
|  | Low | High | **Total** | **p-value** |
| **MCP counter clusters** |  |  |  | 0.2 |
| Immune Low | 27 (77%) | 25 (66%) | 52 (71%) |  |
| Stromal | 4 (11%) | 10 (26%) | 14 (19%) |  |
| Immune High | 4 (11%) | 3 (7.9%) | 7 (9.6%) |  |
| **Total** | 35 (100%) | 38 (100%) | 73 (100%) |  |

Supplementary Table 6: ISIC classes and IHC infiltration markers in the retrospective cohort

| **Characteristic** | **ISIC Low** N = 35 | **ISIC High** N = 38 | **p-value** |
| --- | --- | --- | --- |
| **CD3 Density (CT)** |  |  | <0.001 |
| Mean (SD) | 328 (146) | 532 (296) |  |
| Median (Q1 - Q3) | 293 (239 - 387) | 425 (321 - 733) |  |
| Min - Max | 81 - 619 | 46 - 1,322 |  |
| Unknown | 4 | 1 |  |
| **CD3 Density (IM)** |  |  | 0.010 |
| Mean (SD) | 678 (218) | 961 (450) |  |
| Median (Q1 - Q3) | 657 (487 - 836) | 889 (549 - 1,263) |  |
| Min - Max | 371 - 1,133 | 305 - 2,033 |  |
| Unknown | 4 | 1 |  |
| **CD8 Density (CT)** |  |  | <0.001 |
| Mean (SD) | 73 (36) | 229 (188) |  |
| Median (Q1 - Q3) | 62 (46 - 104) | 145 (97 - 311) |  |
| Min - Max | 26 - 182 | 16 - 735 |  |
| Unknown | 4 | 1 |  |
| **CD8 Density (IM)** |  |  | <0.001 |
| Mean (SD) | 215 (89) | 445 (273) |  |
| Median (Q1 - Q3) | 208 (142 - 267) | 349 (266 - 620) |  |
| Min - Max | 89 - 415 | 71 - 1,187 |  |
| Unknown | 4 | 1 |  |
| **CD68 Density (CT)** |  |  | 0.009 |
| Mean (SD) | 46 (34) | 104 (108) |  |
| Median (Q1 - Q3) | 37 (27 - 59) | 62 (40 - 132) |  |
| Min - Max | 3 - 162 | 8 - 443 |  |
| Unknown | 12 | 10 |  |
| **CD68 Density (IM)** |  |  | 0.5 |
| Mean (SD) | 72 (119) | 108 (186) |  |
| Median (Q1 - Q3) | 32 (18 - 70) | 53 (18 - 92) |  |
| Min - Max | 1 - 472 | 4 - 870 |  |
| Unknown | 12 | 10 |  |

Supplementary Table 7: ISIC Groups and immune cell composition in the retrospective cohort

| **Characteristic** | **ISIC Low** N = 35 | **ISIC High** N = 38 | **p-value** |
| --- | --- | --- | --- |
| **T cells** |  |  | 0.4 |
| Mean (SD) | 1.58 (0.34) | 1.63 (0.32) |  |
| Median (Q1 - Q3) | 1.52 (1.35 - 1.72) | 1.58 (1.41 - 1.81) |  |
| Min - Max | 1.10 - 2.47 | 1.16 - 2.63 |  |
| **CD8 T cells** |  |  | 0.7 |
| Mean (SD) | 0.53 (0.68) | 0.61 (0.74) |  |
| Median (Q1 - Q3) | 0.25 (0.00 - 0.80) | 0.54 (0.01 - 0.86) |  |
| Min - Max | -0.22 - 2.67 | -0.19 - 3.34 |  |
| **Cytotoxic lymphocytes** |  |  | 0.015 |
| Mean (SD) | 1.39 (0.40) | 1.60 (0.49) |  |
| Median (Q1 - Q3) | 1.30 (1.12 - 1.63) | 1.47 (1.33 - 1.78) |  |
| Min - Max | 0.90 - 3.11 | 0.93 - 3.65 |  |
| **B lineage** |  |  | 0.6 |
| Mean (SD) | 1.19 (0.47) | 1.31 (0.64) |  |
| Median (Q1 - Q3) | 1.15 (0.84 - 1.43) | 1.25 (0.88 - 1.49) |  |
| Min - Max | 0.34 - 2.20 | 0.30 - 3.25 |  |
| **NK cells** |  |  | 0.9 |
| Mean (SD) | -0.18 (0.47) | -0.20 (0.43) |  |
| Median (Q1 - Q3) | -0.28 (-0.63 - 0.15) | -0.23 (-0.56 - 0.10) |  |
| Min - Max | -0.84 - 1.19 | -0.83 - 0.91 |  |
| **Monocytic lineage** |  |  | 0.3 |
| Mean (SD) | 3.24 (0.47) | 3.30 (0.36) |  |
| Median (Q1 - Q3) | 3.19 (2.87 - 3.47) | 3.27 (3.05 - 3.50) |  |
| Min - Max | 2.35 - 4.28 | 2.54 - 4.17 |  |
| **Myeloid dendritic cells** |  |  | 0.7 |
| Mean (SD) | 0.12 (0.29) | 0.08 (0.22) |  |
| Median (Q1 - Q3) | 0.07 (-0.10 - 0.31) | 0.02 (-0.10 - 0.22) |  |
| Min - Max | -0.33 - 0.82 | -0.20 - 0.68 |  |
| **Neutrophils** |  |  | 0.8 |
| Mean (SD) | 1.99 (0.36) | 2.00 (0.38) |  |
| Median (Q1 - Q3) | 2.01 (1.73 - 2.19) | 1.89 (1.74 - 2.24) |  |
| Min - Max | 1.18 - 2.84 | 1.44 - 2.93 |  |
| **Endothelial cells** |  |  | 0.6 |
| Mean (SD) | 2.58 (0.29) | 2.57 (0.29) |  |
| Median (Q1 - Q3) | 2.57 (2.34 - 2.85) | 2.53 (2.41 - 2.64) |  |
| Min - Max | 1.94 - 3.11 | 2.00 - 3.62 |  |
| **Fibroblasts** |  |  | >0.9 |
| Mean (SD) | 6.82 (0.84) | 6.85 (0.94) |  |
| Median (Q1 - Q3) | 6.72 (6.04 - 7.54) | 6.79 (6.20 - 7.66) |  |
| Min - Max | 5.37 - 8.38 | 5.44 - 9.32 |  |

Supplementary Table 8: TuLIS-like score and IHC infiltration markers in the retrospective cohort

| **Characteristic** | **TuLIS-like Low** N = 115 | **TuLIS-like High** N = 39 | **p-value** |
| --- | --- | --- | --- |
| **CD3 Density (CT)** |  |  | <0.001 |
| Mean (SD) | 367 (197) | 741 (330) |  |
| Median (Q1 - Q3) | 334 (230 - 478) | 663 (529 - 847) |  |
| Min - Max | 46 - 951 | 298 - 2,127 |  |
| **CD3 Density (IM)** |  |  | <0.001 |
| Mean (SD) | 669 (232) | 1,424 (307) |  |
| Median (Q1 - Q3) | 684 (469 - 836) | 1,376 (1,219 - 1,555) |  |
| Min - Max | 171 - 1,099 | 1,100 - 2,409 |  |
| **CD8 Density (CT)** |  |  | <0.001 |
| Mean (SD) | 113 (88) | 288 (173) |  |
| Median (Q1 - Q3) | 91 (57 - 137) | 263 (152 - 364) |  |
| Min - Max | 16 - 470 | 67 - 735 |  |
| **CD8 Density (IM)** |  |  | <0.001 |
| Mean (SD) | 249 (131) | 632 (234) |  |
| Median (Q1 - Q3) | 229 (144 - 320) | 608 (490 - 807) |  |
| Min - Max | 50 - 659 | 147 - 1,187 |  |
| **CD68 Density (CT)** |  |  | <0.001 |
| Mean (SD) | 185 (229) | 348 (274) |  |
| Median (Q1 - Q3) | 109 (48 - 225) | 332 (146 - 473) |  |
| Min - Max | 3 - 1,615 | 34 - 1,236 |  |
| Unknown | 16 | 6 |  |
| **CD68 Density (IM)** |  |  | 0.11 |
| Mean (SD) | 206 (415) | 291 (516) |  |
| Median (Q1 - Q3) | 70 (27 - 218) | 129 (48 - 267) |  |
| Min - Max | 1 - 3,368 | 5 - 2,627 |  |
| Unknown | 16 | 6 |  |

Supplementary Table 9: TuLIS-like score and MCP counter clusters in the retrospective cohort

|  | **TuLIS-like** | |  |  |
| --- | --- | --- | --- | --- |
|  | Low | High | **Total** | **p-value** |
| **MCP counter clusters** |  |  |  | 0.11 |
| Immune Low | 73 (65%) | 18 (50%) | 91 (61%) |  |
| Stromal | 23 (21%) | 14 (39%) | 37 (25%) |  |
| Immune High | 16 (14%) | 4 (11%) | 20 (14%) |  |
| **Total** | 112 (100%) | 36 (100%) | 148 (100%) |  |

Supplementary Table 10: TuLIS-like score and MCP counter immune cell composition in the retrospective cohort

| **Characteristic** | **TuLIS-like Low** N = 115 | **TuLIS-like High** N = 39 | **p-value** |
| --- | --- | --- | --- |
| **T cells** |  |  | 0.029 |
| Mean (SD) | 1.61 (0.39) | 1.76 (0.32) |  |
| Median (Q1 - Q3) | 1.57 (1.37 - 1.86) | 1.75 (1.55 - 2.05) |  |
| Min - Max | 0.22 - 2.63 | 1.16 - 2.60 |  |
| Unknown | 3 | 3 |  |
| **CD8 T cells** |  |  | 0.2 |
| Mean (SD) | 0.64 (0.73) | 0.45 (0.57) |  |
| Median (Q1 - Q3) | 0.55 (0.06 - 1.00) | 0.30 (0.00 - 0.84) |  |
| Min - Max | -0.41 - 4.18 | -0.37 - 1.99 |  |
| Unknown | 3 | 3 |  |
| **Cytotoxic lymphocytes** |  |  | 0.018 |
| Mean (SD) | 1.61 (0.48) | 1.78 (0.42) |  |
| Median (Q1 - Q3) | 1.51 (1.24 - 1.83) | 1.74 (1.51 - 1.99) |  |
| Min - Max | 0.86 - 3.65 | 0.90 - 2.72 |  |
| Unknown | 3 | 3 |  |
| **B lineage** |  |  | 0.093 |
| Mean (SD) | 1.31 (0.64) | 1.55 (0.73) |  |
| Median (Q1 - Q3) | 1.25 (0.86 - 1.68) | 1.40 (1.06 - 1.90) |  |
| Min - Max | 0.12 - 4.65 | 0.56 - 3.30 |  |
| Unknown | 3 | 3 |  |
| **NK cells** |  |  | 0.7 |
| Mean (SD) | -0.17 (0.46) | -0.16 (0.40) |  |
| Median (Q1 - Q3) | -0.22 (-0.56 - 0.13) | -0.22 (-0.49 - 0.16) |  |
| Min - Max | -0.89 - 1.29 | -0.79 - 0.76 |  |
| Unknown | 3 | 3 |  |
| **Monocytic lineage** |  |  | 0.024 |
| Mean (SD) | 3.22 (0.51) | 3.41 (0.46) |  |
| Median (Q1 - Q3) | 3.22 (2.91 - 3.44) | 3.39 (3.10 - 3.71) |  |
| Min - Max | 1.88 - 5.02 | 2.36 - 4.17 |  |
| Unknown | 3 | 3 |  |
| **Myeloid dendritic cells** |  |  | 0.6 |
| Mean (SD) | 0.10 (0.25) | 0.11 (0.24) |  |
| Median (Q1 - Q3) | 0.08 (-0.10 - 0.23) | 0.08 (-0.08 - 0.29) |  |
| Min - Max | -0.33 - 0.82 | -0.38 - 0.64 |  |
| Unknown | 3 | 3 |  |
| **Neutrophils** |  |  | 0.2 |
| Mean (SD) | 2.05 (0.41) | 2.13 (0.39) |  |
| Median (Q1 - Q3) | 2.00 (1.76 - 2.27) | 2.08 (1.87 - 2.33) |  |
| Min - Max | 1.18 - 3.65 | 1.44 - 3.22 |  |
| Unknown | 3 | 3 |  |
| **Endothelial cells** |  |  | 0.3 |
| Mean (SD) | 2.63 (0.36) | 2.57 (0.28) |  |
| Median (Q1 - Q3) | 2.61 (2.36 - 2.87) | 2.52 (2.39 - 2.73) |  |
| Min - Max | 1.38 - 3.62 | 2.11 - 3.22 |  |
| Unknown | 3 | 3 |  |
| **Fibroblasts** |  |  | 0.5 |
| Mean (SD) | 6.84 (0.98) | 6.96 (0.91) |  |
| Median (Q1 - Q3) | 6.76 (6.04 - 7.50) | 7.01 (6.44 - 7.71) |  |
| Min - Max | 4.19 - 9.32 | 5.20 - 8.32 |  |
| Unknown | 3 | 3 |  |

Supplementary Table 11: TuLIS-like score and ISIC classes in the retrospective cohort

|  | **TuLIS-like** | |  |  |
| --- | --- | --- | --- | --- |
|  | Low | High | **Total** | **p-value** |
| **ISIC classes** |  |  |  | 0.008 |
| Low | 29 (54%) | 2 (14%) | 31 (46%) |  |
| High | 25 (46%) | 12 (86%) | 37 (54%) |  |
| **Total** | 54 (100%) | 14 (100%) | 68 (100%) |  |

Supplementary Table 12: RAS Mutated variant and IHC infiltration markers in the retrospective cohort

| **Characteristic** | **KRAS G12D** N = 35 | **KRAS G12V** N = 20 | **KRAS G13D** N = 19 | **KRAS G12C** N = 13 | **Other KRAS** N = 58 | **NRAS** N = 15 | **p-value** |
| --- | --- | --- | --- | --- | --- | --- | --- |
| **CD3 Density (CT)** |  |  |  |  |  |  | 0.8 |
| Mean (SD) | 477 (261) | 574 (486) | 400 (233) | 469 (184) | 442 (268) | 440 (193) |  |
| Median (Q1 - Q3) | 395 (305 - 612) | 418 (262 - 663) | 371 (220 - 515) | 441 (299 - 605) | 397 (236 - 600) | 420 (279 - 643) |  |
| Min - Max | 151 - 1,322 | 46 - 2,127 | 67 - 878 | 250 - 795 | 58 - 1,124 | 179 - 806 |  |
| Unknown | 2 | 1 | 1 | 1 | 1 | 1 |  |
| **CD3 Density (IM)** |  |  |  |  |  |  | 0.6 |
| Mean (SD) | 874 (425) | 1,026 (652) | 705 (330) | 879 (242) | 846 (377) | 861 (328) |  |
| Median (Q1 - Q3) | 808 (612 - 1,100) | 861 (587 - 1,524) | 602 (442 - 949) | 828 (744 - 900) | 785 (543 - 1,110) | 778 (638 - 938) |  |
| Min - Max | 223 - 2,033 | 171 - 2,409 | 228 - 1,265 | 644 - 1,555 | 201 - 1,677 | 460 - 1,558 |  |
| Unknown | 2 | 1 | 1 | 1 | 1 | 1 |  |
| **CD8 Density (CT)** |  |  |  |  |  |  | >0.9 |
| Mean (SD) | 174 (174) | 163 (138) | 128 (99) | 150 (91) | 164 (147) | 131 (79) |  |
| Median (Q1 - Q3) | 111 (58 - 217) | 109 (80 - 263) | 103 (62 - 154) | 124 (86 - 206) | 101 (69 - 232) | 112 (79 - 169) |  |
| Min - Max | 26 - 722 | 19 - 546 | 21 - 404 | 53 - 311 | 16 - 735 | 28 - 308 |  |
| Unknown | 2 | 1 | 1 | 1 | 1 | 1 |  |
| **CD8 Density (IM)** |  |  |  |  |  |  | >0.9 |
| Mean (SD) | 342 (271) | 350 (251) | 299 (164) | 343 (126) | 371 (258) | 322 (167) |  |
| Median (Q1 - Q3) | 267 (131 - 404) | 267 (152 - 546) | 266 (161 - 438) | 329 (276 - 428) | 286 (170 - 531) | 321 (192 - 491) |  |
| Min - Max | 50 - 1,187 | 61 - 1,045 | 90 - 597 | 168 - 625 | 71 - 955 | 85 - 630 |  |
| Unknown | 2 | 1 | 1 | 1 | 1 | 1 |  |
| **CD68 Density (CT)** |  |  |  |  |  |  | 0.5 |
| Mean (SD) | 202 (217) | 295 (344) | 186 (242) | 187 (194) | 245 (283) | 223 (193) |  |
| Median (Q1 - Q3) | 110 (43 - 285) | 159 (67 - 475) | 74 (29 - 261) | 113 (53 - 300) | 145 (62 - 337) | 176 (74 - 279) |  |
| Min - Max | 3 - 834 | 35 - 1,236 | 9 - 817 | 8 - 539 | 13 - 1,615 | 30 - 644 |  |
| Unknown | 11 | 3 | 3 | 4 | 3 | 1 |  |
| **CD68 Density (IM)** |  |  |  |  |  |  | 0.013 |
| Mean (SD) | 118 (317) | 149 (212) | 155 (208) | 331 (620) | 342 (630) | 207 (182) |  |
| Median (Q1 - Q3) | 52 (20 - 83) | 69 (33 - 147) | 53 (25 - 216) | 86 (24 - 97) | 140 (48 - 351) | 136 (59 - 328) |  |
| Min - Max | 1 - 1,593 | 7 - 870 | 2 - 713 | 5 - 1,860 | 5 - 3,368 | 31 - 572 |  |
| Unknown | 11 | 3 | 3 | 4 | 3 | 1 |  |

Supplementary Table 13: RAS Mutated variant and MCP counter immune cell composition in the retrospective cohort

| **Characteristic** | **KRAS G12D** N = 35 | **KRAS G12V** N = 20 | **KRAS G13D** N = 19 | **KRAS G12C** N = 13 | **Other KRAS** N = 58 | **NRAS** N = 15 | **p-value** |
| --- | --- | --- | --- | --- | --- | --- | --- |
| **T cells** |  |  |  |  |  |  | 0.6 |
| Mean (SD) | 1.60 (0.32) | 1.58 (0.35) | 1.62 (0.34) | 1.72 (0.33) | 1.70 (0.37) | 1.64 (0.59) |  |
| Median (Q1 - Q3) | 1.55 (1.37 - 1.88) | 1.63 (1.28 - 1.80) | 1.55 (1.42 - 1.78) | 1.71 (1.52 - 1.91) | 1.61 (1.48 - 1.92) | 1.71 (1.40 - 2.07) |  |
| Min - Max | 0.84 - 2.34 | 1.15 - 2.24 | 1.17 - 2.63 | 1.19 - 2.32 | 0.86 - 2.60 | 0.22 - 2.50 |  |
| Unknown | 0 | 0 | 0 | 2 | 2 | 3 |  |
| **CD8 T cells** |  |  |  |  |  |  | 0.6 |
| Mean (SD) | 0.45 (0.50) | 0.62 (0.72) | 0.77 (0.99) | 0.56 (0.78) | 0.59 (0.69) | 0.84 (0.77) |  |
| Median (Q1 - Q3) | 0.32 (-0.02 - 0.77) | 0.44 (0.00 - 1.16) | 0.51 (0.00 - 1.40) | 0.36 (-0.01 - 1.11) | 0.46 (0.13 - 0.81) | 0.74 (0.51 - 1.11) |  |
| Min - Max | -0.12 - 1.91 | -0.25 - 1.99 | -0.34 - 3.34 | -0.41 - 2.05 | -0.11 - 4.18 | -0.37 - 2.27 |  |
| Unknown | 0 | 0 | 0 | 2 | 2 | 3 |  |
| **Cytotoxic lymphocytes** |  |  |  |  |  |  | 0.3 |
| Mean (SD) | 1.66 (0.48) | 1.58 (0.44) | 1.47 (0.32) | 1.47 (0.38) | 1.69 (0.52) | 1.81 (0.53) |  |
| Median (Q1 - Q3) | 1.63 (1.22 - 2.02) | 1.62 (1.30 - 1.78) | 1.39 (1.21 - 1.74) | 1.39 (1.15 - 1.83) | 1.63 (1.32 - 1.90) | 1.78 (1.27 - 2.33) |  |
| Min - Max | 0.92 - 3.11 | 0.93 - 2.72 | 1.06 - 2.29 | 0.99 - 2.26 | 0.86 - 3.65 | 1.02 - 2.39 |  |
| Unknown | 0 | 0 | 0 | 2 | 2 | 3 |  |
| **B lineage** |  |  |  |  |  |  | 0.6 |
| Mean (SD) | 1.33 (0.58) | 1.46 (0.82) | 1.12 (0.45) | 1.29 (0.79) | 1.36 (0.50) | 1.65 (1.21) |  |
| Median (Q1 - Q3) | 1.24 (0.88 - 1.64) | 1.41 (0.78 - 2.06) | 1.11 (0.83 - 1.40) | 1.12 (0.77 - 1.68) | 1.31 (1.02 - 1.72) | 1.24 (1.01 - 2.28) |  |
| Min - Max | 0.44 - 3.30 | 0.30 - 3.21 | 0.34 - 2.09 | 0.32 - 3.25 | 0.27 - 2.49 | 0.12 - 4.65 |  |
| Unknown | 0 | 0 | 0 | 2 | 2 | 3 |  |
| **NK cells** |  |  |  |  |  |  | 0.7 |
| Mean (SD) | -0.17 (0.47) | -0.21 (0.44) | -0.22 (0.39) | -0.35 (0.43) | -0.12 (0.47) | -0.18 (0.41) |  |
| Median (Q1 - Q3) | -0.22 (-0.55 - 0.15) | -0.19 (-0.54 - 0.09) | -0.28 (-0.49 - 0.12) | -0.53 (-0.64 - -0.08) | -0.22 (-0.50 - 0.23) | -0.17 (-0.52 - -0.06) |  |
| Min - Max | -0.78 - 1.19 | -0.83 - 0.62 | -0.89 - 0.61 | -0.70 - 0.63 | -0.79 - 1.29 | -0.79 - 0.59 |  |
| Unknown | 0 | 0 | 0 | 2 | 2 | 3 |  |
| **Monocytic lineage** |  |  |  |  |  |  | 0.8 |
| Mean (SD) | 3.29 (0.46) | 3.32 (0.52) | 3.19 (0.53) | 3.23 (0.45) | 3.29 (0.47) | 3.22 (0.75) |  |
| Median (Q1 - Q3) | 3.28 (2.97 - 3.57) | 3.30 (3.02 - 3.66) | 3.13 (2.87 - 3.37) | 3.19 (2.91 - 3.45) | 3.26 (2.98 - 3.54) | 2.95 (2.81 - 3.83) |  |
| Min - Max | 2.18 - 4.17 | 2.17 - 4.18 | 2.35 - 4.85 | 2.67 - 4.28 | 2.30 - 5.02 | 1.88 - 4.53 |  |
| Unknown | 0 | 0 | 0 | 2 | 2 | 3 |  |
| **Myeloid dendritic cells** |  |  |  |  |  |  | 0.7 |
| Mean (SD) | 0.07 (0.25) | 0.17 (0.29) | 0.11 (0.22) | 0.13 (0.27) | 0.09 (0.24) | 0.12 (0.20) |  |
| Median (Q1 - Q3) | 0.04 (-0.10 - 0.24) | 0.18 (-0.09 - 0.38) | 0.11 (-0.05 - 0.25) | 0.08 (-0.17 - 0.37) | 0.06 (-0.09 - 0.16) | 0.18 (-0.01 - 0.25) |  |
| Min - Max | -0.38 - 0.67 | -0.26 - 0.68 | -0.33 - 0.61 | -0.19 - 0.70 | -0.25 - 0.82 | -0.29 - 0.38 |  |
| Unknown | 0 | 0 | 0 | 2 | 2 | 3 |  |
| **Neutrophils** |  |  |  |  |  |  | 0.092 |
| Mean (SD) | 2.10 (0.42) | 2.14 (0.40) | 1.90 (0.47) | 1.90 (0.25) | 2.10 (0.36) | 2.17 (0.50) |  |
| Median (Q1 - Q3) | 2.05 (1.77 - 2.39) | 2.05 (1.87 - 2.35) | 1.82 (1.58 - 2.11) | 1.87 (1.76 - 2.01) | 2.04 (1.88 - 2.29) | 2.10 (1.76 - 2.42) |  |
| Min - Max | 1.41 - 3.15 | 1.60 - 3.01 | 1.18 - 3.22 | 1.54 - 2.52 | 1.44 - 3.65 | 1.63 - 3.20 |  |
| Unknown | 0 | 0 | 0 | 2 | 2 | 3 |  |
| **Endothelial cells** |  |  |  |  |  |  | 0.6 |
| Mean (SD) | 2.68 (0.37) | 2.58 (0.28) | 2.54 (0.24) | 2.47 (0.30) | 2.63 (0.32) | 2.60 (0.56) |  |
| Median (Q1 - Q3) | 2.63 (2.43 - 2.86) | 2.60 (2.34 - 2.73) | 2.53 (2.36 - 2.67) | 2.53 (2.19 - 2.72) | 2.56 (2.41 - 2.88) | 2.68 (2.27 - 2.99) |  |
| Min - Max | 2.00 - 3.62 | 2.22 - 3.19 | 2.12 - 3.06 | 1.94 - 2.93 | 1.99 - 3.34 | 1.38 - 3.52 |  |
| Unknown | 0 | 0 | 0 | 2 | 2 | 3 |  |
| **Fibroblasts** |  |  |  |  |  |  | 0.8 |
| Mean (SD) | 7.05 (1.06) | 6.85 (1.00) | 6.67 (0.82) | 7.06 (1.02) | 6.85 (0.87) | 6.70 (1.17) |  |
| Median (Q1 - Q3) | 6.86 (6.17 - 7.90) | 6.66 (5.98 - 7.86) | 6.69 (6.01 - 7.27) | 6.96 (6.09 - 7.87) | 6.84 (6.20 - 7.56) | 6.71 (5.96 - 7.47) |  |
| Min - Max | 5.20 - 9.32 | 5.46 - 8.30 | 5.37 - 8.28 | 5.44 - 8.91 | 4.41 - 8.68 | 4.19 - 8.74 |  |
| Unknown | 0 | 0 | 0 | 2 | 2 | 3 |  |

Supplementary Table 14: Mutation count and IHC markers in the retrospective cohort

| **Characteristic** | **Mutation count <= Median** N = 46 | **Mutation count > Median** N = 42 | **p-value** |
| --- | --- | --- | --- |
| **CD3 Density (CT)** |  |  | 0.2 |
| Mean (SD) | 398 (203) | 512 (315) |  |
| Median (Q1 - Q3) | 379 (269 - 496) | 413 (256 - 729) |  |
| Min - Max | 46 - 1,124 | 81 - 1,322 |  |
| Unknown | 3 | 2 |  |
| **CD3 Density (IM)** |  |  | 0.015 |
| Mean (SD) | 737 (354) | 966 (474) |  |
| Median (Q1 - Q3) | 694 (463 - 892) | 869 (628 - 1,172) |  |
| Min - Max | 272 - 1,677 | 371 - 2,409 |  |
| Unknown | 3 | 2 |  |
| **CD8 Density (CT)** |  |  | 0.014 |
| Mean (SD) | 119 (118) | 199 (175) |  |
| Median (Q1 - Q3) | 92 (58 - 119) | 118 (93 - 289) |  |
| Min - Max | 16 - 735 | 29 - 722 |  |
| Unknown | 3 | 2 |  |
| **CD8 Density (IM)** |  |  | 0.001 |
| Mean (SD) | 259 (176) | 420 (269) |  |
| Median (Q1 - Q3) | 201 (118 - 319) | 312 (236 - 555) |  |
| Min - Max | 71 - 774 | 118 - 1,187 |  |
| Unknown | 3 | 2 |  |
| **CD68 Density (CT)** |  |  | 0.15 |
| Mean (SD) | 159 (208) | 180 (229) |  |
| Median (Q1 - Q3) | 52 (28 - 225) | 66 (48 - 162) |  |
| Min - Max | 3 - 817 | 25 - 771 |  |
| Unknown | 9 | 13 |  |
| **CD68 Density (IM)** |  |  | 0.12 |
| Mean (SD) | 95 (150) | 140 (191) |  |
| Median (Q1 - Q3) | 50 (18 - 97) | 70 (31 - 154) |  |
| Min - Max | 1 - 753 | 5 - 870 |  |
| Unknown | 9 | 13 |  |

Supplementary Table 15: Mutation count and MCP counter immune cell composition in the retrospective cohort

| **Characteristic** | **Mutation count <= Median** N = 46 | **Mutation count > Median** N = 42 | **p-value** |
| --- | --- | --- | --- |
| **T cells** |  |  | 0.5 |
| Mean (SD) | 1.61 (0.31) | 1.66 (0.35) |  |
| Median (Q1 - Q3) | 1.56 (1.37 - 1.85) | 1.59 (1.42 - 1.81) |  |
| Min - Max | 1.10 - 2.32 | 0.84 - 2.63 |  |
| **CD8 T cells** |  |  | 0.3 |
| Mean (SD) | 0.50 (0.66) | 0.61 (0.67) |  |
| Median (Q1 - Q3) | 0.29 (-0.01 - 0.71) | 0.44 (0.18 - 0.86) |  |
| Min - Max | -0.41 - 2.67 | -0.25 - 3.34 |  |
| **Cytotoxic lymphocytes** |  |  | 0.5 |
| Mean (SD) | 1.60 (0.54) | 1.50 (0.42) |  |
| Median (Q1 - Q3) | 1.46 (1.22 - 1.75) | 1.39 (1.15 - 1.75) |  |
| Min - Max | 0.93 - 3.65 | 0.90 - 2.72 |  |
| **B lineage** |  |  | 0.9 |
| Mean (SD) | 1.33 (0.64) | 1.34 (0.57) |  |
| Median (Q1 - Q3) | 1.31 (0.84 - 1.64) | 1.23 (0.90 - 1.75) |  |
| Min - Max | 0.30 - 3.25 | 0.32 - 3.21 |  |
| **NK cells** |  |  | 0.7 |
| Mean (SD) | -0.18 (0.43) | -0.16 (0.44) |  |
| Median (Q1 - Q3) | -0.28 (-0.49 - 0.12) | -0.21 (-0.55 - 0.18) |  |
| Min - Max | -0.84 - 1.19 | -0.79 - 0.91 |  |
| **Monocytic lineage** |  |  | 0.4 |
| Mean (SD) | 3.31 (0.49) | 3.36 (0.46) |  |
| Median (Q1 - Q3) | 3.25 (2.93 - 3.50) | 3.36 (3.03 - 3.63) |  |
| Min - Max | 2.35 - 4.85 | 2.18 - 4.28 |  |
| **Myeloid dendritic cells** |  |  | 0.8 |
| Mean (SD) | 0.13 (0.24) | 0.15 (0.29) |  |
| Median (Q1 - Q3) | 0.08 (-0.06 - 0.25) | 0.08 (-0.08 - 0.35) |  |
| Min - Max | -0.22 - 0.68 | -0.33 - 0.82 |  |
| **Neutrophils** |  |  | 0.8 |
| Mean (SD) | 2.08 (0.42) | 2.07 (0.41) |  |
| Median (Q1 - Q3) | 2.04 (1.76 - 2.28) | 1.99 (1.80 - 2.29) |  |
| Min - Max | 1.18 - 3.15 | 1.44 - 3.22 |  |
| **Endothelial cells** |  |  | 0.4 |
| Mean (SD) | 2.65 (0.34) | 2.58 (0.31) |  |
| Median (Q1 - Q3) | 2.62 (2.42 - 2.86) | 2.55 (2.35 - 2.81) |  |
| Min - Max | 2.12 - 3.62 | 1.94 - 3.19 |  |
| **Fibroblasts** |  |  | 0.3 |
| Mean (SD) | 7.09 (1.06) | 6.85 (0.85) |  |
| Median (Q1 - Q3) | 6.98 (6.20 - 7.90) | 6.90 (6.09 - 7.50) |  |
| Min - Max | 5.37 - 9.32 | 5.44 - 8.68 |  |

Supplementary Table 16: Immunoscore and IHC infiltration markers in the ambispective cohort

| **Characteristic** | **IS0-2** N = 98 | **IS3** N = 10 | **p-value** |
| --- | --- | --- | --- |
| **CD3 Density (CT)** |  |  | <0.001 |
| Mean (SD) | 320 (171) | 1,091 (404) |  |
| Median (Q1 - Q3) | 279 (185 - 445) | 997 (769 - 1,411) |  |
| Min - Max | 34 - 761 | 665 - 1,709 |  |
| Unknown | 19 | 4 |  |
| **CD3 Density (IM)** |  |  | <0.001 |
| Mean (SD) | 602 (283) | 1,569 (374) |  |
| Median (Q1 - Q3) | 579 (394 - 792) | 1,498 (1,219 - 1,932) |  |
| Min - Max | 103 - 1,310 | 1,217 - 2,050 |  |
| Unknown | 19 | 4 |  |
| **CD8 Density (CT)** |  |  | <0.001 |
| Mean (SD) | 107 (68) | 532 (134) |  |
| Median (Q1 - Q3) | 84 (60 - 140) | 507 (480 - 601) |  |
| Min - Max | 25 - 354 | 351 - 749 |  |
| Unknown | 19 | 4 |  |
| **CD8 Density (IM)** |  |  | <0.001 |
| Mean (SD) | 249 (135) | 815 (238) |  |
| Median (Q1 - Q3) | 225 (141 - 337) | 771 (681 - 968) |  |
| Min - Max | 33 - 596 | 514 - 1,183 |  |
| Unknown | 19 | 4 |  |
| **CD68 Density (CT)** |  |  | 0.038 |
| Mean (SD) | 156 (214) | 325 (320) |  |
| Median (Q1 - Q3) | 105 (35 - 199) | 204 (110 - 545) |  |
| Min - Max | 6 - 1,539 | 19 - 969 |  |
| Unknown | 2 | 0 |  |
| **CD68 Density (IM)** |  |  | 0.4 |
| Mean (SD) | 63 (59) | 74 (45) |  |
| Median (Q1 - Q3) | 41 (23 - 87) | 94 (25 - 117) |  |
| Min - Max | 0 - 248 | 23 - 124 |  |
| Unknown | 9 | 3 |  |

Supplementary Table 17: Immunoscore in MCP counter immune cell composition in the ambispective cohort

| **Characteristic** | **IS0-2** N = 98 | **IS3** N = 10 | **p-value** |
| --- | --- | --- | --- |
| **T cells** |  |  | 0.079 |
| Mean (SD) | 0.78 (0.75) | 1.02 (0.54) |  |
| Median (Q1 - Q3) | 0.56 (0.35 - 0.93) | 1.02 (0.63 - 1.31) |  |
| Min - Max | -0.54 - 3.04 | 0.16 - 2.03 |  |
| Unknown | 2 | 0 |  |
| **CD8 T cells** |  |  | 0.9 |
| Mean (SD) | -0.37 (1.13) | -0.61 (0.66) |  |
| Median (Q1 - Q3) | -0.59 (-1.20 - 0.02) | -0.67 (-1.17 - -0.13) |  |
| Min - Max | -1.36 - 3.68 | -1.35 - 0.64 |  |
| Unknown | 2 | 0 |  |
| **Cytotoxic lymphocytes** |  |  | 0.025 |
| Mean (SD) | 0.89 (0.87) | 1.51 (0.76) |  |
| Median (Q1 - Q3) | 0.73 (0.34 - 1.42) | 1.58 (1.09 - 2.07) |  |
| Min - Max | -0.79 - 4.00 | 0.14 - 2.39 |  |
| Unknown | 2 | 0 |  |
| **B lineage** |  |  | >0.9 |
| Mean (SD) | 0.74 (0.86) | 0.76 (0.80) |  |
| Median (Q1 - Q3) | 0.59 (0.28 - 0.94) | 0.72 (0.25 - 0.90) |  |
| Min - Max | -0.83 - 3.96 | -0.14 - 2.36 |  |
| Unknown | 2 | 0 |  |
| **NK cells** |  |  | 0.2 |
| Mean (SD) | -0.15 (0.67) | -0.43 (0.44) |  |
| Median (Q1 - Q3) | -0.29 (-0.55 - 0.18) | -0.42 (-0.81 - -0.16) |  |
| Min - Max | -1.02 - 2.44 | -1.02 - 0.30 |  |
| Unknown | 2 | 0 |  |
| **Monocytic lineage** |  |  | 0.2 |
| Mean (SD) | 2.21 (0.93) | 2.71 (1.05) |  |
| Median (Q1 - Q3) | 2.08 (1.53 - 2.96) | 2.65 (1.71 - 3.86) |  |
| Min - Max | -0.03 - 4.13 | 1.39 - 4.16 |  |
| Unknown | 2 | 0 |  |
| **Myeloid dendritic cells** |  |  | >0.9 |
| Mean (SD) | 0.14 (1.14) | -0.13 (0.43) |  |
| Median (Q1 - Q3) | -0.19 (-0.49 - 0.26) | -0.09 (-0.45 - 0.13) |  |
| Min - Max | -0.97 - 6.11 | -0.75 - 0.64 |  |
| Unknown | 2 | 0 |  |
| **Neutrophils** |  |  | 0.4 |
| Mean (SD) | 1.20 (0.53) | 1.07 (0.35) |  |
| Median (Q1 - Q3) | 1.10 (0.93 - 1.31) | 0.98 (0.80 - 1.25) |  |
| Min - Max | -0.16 - 2.88 | 0.61 - 1.70 |  |
| Unknown | 2 | 0 |  |
| **Endothelial cells** |  |  | 0.8 |
| Mean (SD) | 1.51 (0.75) | 1.41 (0.53) |  |
| Median (Q1 - Q3) | 1.42 (1.13 - 1.75) | 1.48 (0.93 - 1.76) |  |
| Min - Max | -0.29 - 3.61 | 0.64 - 2.30 |  |
| Unknown | 2 | 0 |  |
| **Fibroblasts** |  |  | 0.8 |
| Mean (SD) | 6.81 (1.19) | 6.85 (1.29) |  |
| Median (Q1 - Q3) | 6.41 (5.75 - 7.88) | 7.35 (5.42 - 7.98) |  |
| Min - Max | 4.94 - 9.07 | 5.17 - 8.39 |  |
| Unknown | 2 | 0 |  |

Supplementary Table 18: MCP Counter clusters and immune cell composition in the ambispective cohort

| **Characteristic** | **Immune Low** N = 73 | **Stromal** N = 34 | **Immune High** N = 12 | **p-value** |
| --- | --- | --- | --- | --- |
| **T cells** |  |  |  | <0.001 |
| Mean (SD) | 0.47 (0.39) | 1.06 (0.63) | 2.49 (0.51) |  |
| Median (Q1 - Q3) | 0.43 (0.24 - 0.64) | 0.89 (0.64 - 1.46) | 2.57 (2.27 - 2.90) |  |
| Min - Max | -0.54 - 1.62 | 0.15 - 3.03 | 1.56 - 3.04 |  |
| **CD8 T cells** |  |  |  | <0.001 |
| Mean (SD) | -0.43 (0.75) | -1.07 (0.32) | 1.98 (1.24) |  |
| Median (Q1 - Q3) | -0.48 (-1.15 - 0.02) | -1.16 (-1.35 - -0.98) | 2.25 (1.79 - 2.48) |  |
| Min - Max | -1.36 - 2.94 | -1.36 - 0.02 | -0.60 - 3.68 |  |
| **Cytotoxic lymphocytes** |  |  |  | <0.001 |
| Mean (SD) | 0.57 (0.63) | 1.19 (0.61) | 2.76 (0.54) |  |
| Median (Q1 - Q3) | 0.50 (0.18 - 0.89) | 1.26 (0.84 - 1.49) | 2.81 (2.28 - 2.95) |  |
| Min - Max | -0.79 - 2.34 | -0.31 - 2.39 | 2.12 - 4.00 |  |
| **B lineage** |  |  |  | <0.001 |
| Mean (SD) | 0.44 (0.59) | 0.90 (0.66) | 2.70 (0.74) |  |
| Median (Q1 - Q3) | 0.39 (0.10 - 0.73) | 0.77 (0.54 - 1.15) | 2.78 (2.50 - 3.05) |  |
| Min - Max | -0.83 - 2.96 | -0.04 - 2.45 | 0.80 - 3.96 |  |
| **NK cells** |  |  |  | <0.001 |
| Mean (SD) | -0.32 (0.48) | -0.27 (0.55) | 1.16 (0.79) |  |
| Median (Q1 - Q3) | -0.42 (-0.63 - 0.12) | -0.33 (-0.57 - 0.09) | 1.32 (1.08 - 1.54) |  |
| Min - Max | -1.02 - 0.90 | -1.01 - 1.19 | -0.40 - 2.44 |  |
| **Monocytic lineage** |  |  |  | <0.001 |
| Mean (SD) | 1.69 (0.55) | 3.09 (0.61) | 3.57 (0.55) |  |
| Median (Q1 - Q3) | 1.66 (1.44 - 2.04) | 3.02 (2.59 - 3.65) | 3.54 (3.27 - 3.69) |  |
| Min - Max | -0.03 - 3.01 | 1.96 - 4.16 | 2.72 - 4.98 |  |
| **Myeloid dendritic cells** |  |  |  | <0.001 |
| Mean (SD) | -0.16 (0.54) | -0.11 (0.48) | 2.98 (1.31) |  |
| Median (Q1 - Q3) | -0.32 (-0.50 - 0.18) | -0.15 (-0.44 - 0.22) | 2.96 (2.44 - 3.47) |  |
| Min - Max | -0.97 - 1.76 | -0.99 - 0.99 | 0.80 - 6.11 |  |
| **Neutrophils** |  |  |  | <0.001 |
| Mean (SD) | 1.03 (0.35) | 1.24 (0.36) | 2.42 (0.39) |  |
| Median (Q1 - Q3) | 1.06 (0.83 - 1.20) | 1.18 (0.91 - 1.45) | 2.46 (2.10 - 2.79) |  |
| Min - Max | -0.16 - 1.82 | 0.70 - 2.19 | 1.81 - 2.91 |  |
| **Endothelial cells** |  |  |  | <0.001 |
| Mean (SD) | 1.20 (0.51) | 1.67 (0.43) | 3.27 (0.43) |  |
| Median (Q1 - Q3) | 1.25 (0.97 - 1.50) | 1.66 (1.36 - 1.89) | 3.23 (2.97 - 3.51) |  |
| Min - Max | -0.29 - 2.40 | 0.73 - 2.65 | 2.62 - 4.23 |  |
| **Fibroblasts** |  |  |  | <0.001 |
| Mean (SD) | 6.32 (0.96) | 8.07 (0.79) | 6.00 (0.92) |  |
| Median (Q1 - Q3) | 6.01 (5.60 - 7.20) | 8.16 (7.81 - 8.50) | 5.71 (5.62 - 5.97) |  |
| Min - Max | 4.71 - 8.54 | 5.50 - 9.07 | 5.37 - 8.87 |  |

Supplementary Table 19: RAS Mutated variant and IHC infiltration markers in the ambispective cohort

| **Characteristic** | **KRAS G12D** N = 23 | **KRAS G12V** N = 16 | **KRAS G13D** N = 11 | **KRAS G12C** N = 5 | **Other KRAS** N = 14 | **NRAS** N = 6 | **p-value** |
| --- | --- | --- | --- | --- | --- | --- | --- |
| **CD3 Density (CT)** |  |  |  |  |  |  | 0.3 |
| Mean (SD) | 296 (186) | 436 (432) | 430 (206) | 331 (278) | 410 (362) | 547 (279) |  |
| Median (Q1 - Q3) | 271 (155 - 412) | 277 (159 - 493) | 343 (283 - 561) | 272 (102 - 429) | 358 (185 - 460) | 651 (290 - 692) |  |
| Min - Max | 53 - 769 | 117 - 1,709 | 160 - 735 | 90 - 761 | 34 - 1,411 | 224 - 877 |  |
| Unknown | 0 | 1 | 2 | 0 | 2 | 1 |  |
| **CD3 Density (IM)** |  |  |  |  |  |  | 0.6 |
| Mean (SD) | 599 (372) | 720 (600) | 807 (273) | 745 (259) | 737 (384) | 726 (380) |  |
| Median (Q1 - Q3) | 507 (325 - 922) | 504 (307 - 971) | 792 (651 - 1,006) | 838 (536 - 951) | 756 (460 - 1,049) | 682 (491 - 978) |  |
| Min - Max | 163 - 1,697 | 109 - 2,050 | 431 - 1,242 | 409 - 991 | 103 - 1,300 | 262 - 1,217 |  |
| Unknown | 0 | 1 | 2 | 0 | 2 | 1 |  |
| **CD8 Density (CT)** |  |  |  |  |  |  | 0.8 |
| Mean (SD) | 126 (121) | 158 (205) | 148 (76) | 103 (69) | 158 (140) | 216 (190) |  |
| Median (Q1 - Q3) | 90 (58 - 150) | 73 (60 - 180) | 142 (116 - 186) | 85 (58 - 125) | 100 (52 - 208) | 124 (62 - 354) |  |
| Min - Max | 25 - 601 | 33 - 749 | 31 - 286 | 36 - 212 | 26 - 484 | 60 - 480 |  |
| Unknown | 0 | 1 | 2 | 0 | 2 | 1 |  |
| **CD8 Density (IM)** |  |  |  |  |  |  | 0.5 |
| Mean (SD) | 277 (233) | 298 (276) | 356 (152) | 323 (116) | 307 (192) | 309 (262) |  |
| Median (Q1 - Q3) | 222 (118 - 326) | 210 (132 - 306) | 337 (259 - 491) | 364 (251 - 409) | 311 (148 - 422) | 169 (168 - 456) |  |
| Min - Max | 69 - 1,183 | 33 - 968 | 136 - 596 | 157 - 435 | 60 - 681 | 56 - 696 |  |
| Unknown | 0 | 1 | 2 | 0 | 2 | 1 |  |
| **CD68 Density (CT)** |  |  |  |  |  |  | 0.5 |
| Mean (SD) | 130 (120) | 169 (262) | 128 (83) | 37 (14) | 180 (244) | 204 (343) |  |
| Median (Q1 - Q3) | 107 (36 - 173) | 54 (21 - 183) | 115 (69 - 201) | 34 (27 - 48) | 112 (36 - 223) | 79 (32 - 110) |  |
| Min - Max | 7 - 495 | 4 - 969 | 14 - 303 | 25 - 56 | 6 - 959 | 21 - 901 |  |
| Unknown | 0 | 1 | 0 | 1 | 0 | 0 |  |
| **CD68 Density (IM)** |  |  |  |  |  |  | 0.3 |
| Mean (SD) | 51 (47) | 58 (62) | 75 (63) | 28 (29) | 39 (38) | 34 (45) |  |
| Median (Q1 - Q3) | 39 (13 - 70) | 26 (23 - 75) | 45 (37 - 93) | 19 (9 - 48) | 25 (11 - 61) | 16 (7 - 39) |  |
| Min - Max | 1 - 151 | 5 - 217 | 17 - 205 | 5 - 70 | 0 - 118 | 6 - 122 |  |
| Unknown | 0 | 3 | 0 | 1 | 1 | 0 |  |

Supplementary Table 20: RAS Mutated variant and MCP counter immune cell composition in the ambispective cohort

| **Characteristic** | **KRAS G12D** N = 23 | **KRAS G12V** N = 16 | **KRAS G13D** N = 11 | **KRAS G12C** N = 5 | **Other KRAS** N = 14 | **NRAS** N = 6 | **p-value** |
| --- | --- | --- | --- | --- | --- | --- | --- |
| **T cells** |  |  |  |  |  |  | 0.4 |
| Mean (SD) | 0.77 (0.69) | 0.55 (0.61) | 1.13 (0.86) | 0.93 (0.69) | 0.77 (0.46) | 1.19 (0.71) |  |
| Median (Q1 - Q3) | 0.60 (0.42 - 1.02) | 0.60 (0.18 - 0.88) | 0.79 (0.56 - 1.48) | 0.69 (0.57 - 1.16) | 0.67 (0.47 - 1.02) | 1.36 (0.47 - 1.54) |  |
| Min - Max | -0.29 - 3.03 | -0.54 - 2.03 | 0.27 - 2.89 | 0.23 - 2.01 | 0.13 - 1.89 | 0.24 - 2.15 |  |
| **CD8 T cells** |  |  |  |  |  |  | 0.2 |
| Mean (SD) | -0.57 (1.03) | -0.93 (0.52) | -0.65 (1.48) | -0.74 (0.62) | -0.82 (0.62) | -1.30 (0.08) |  |
| Median (Q1 - Q3) | -0.77 (-1.28 - -0.16) | -1.07 (-1.33 - -0.85) | -1.16 (-1.35 - -0.98) | -0.73 (-1.23 - -0.59) | -1.15 (-1.21 - -0.13) | -1.35 (-1.35 - -1.22) |  |
| Min - Max | -1.36 - 3.39 | -1.36 - 0.20 | -1.36 - 3.68 | -1.35 - 0.19 | -1.35 - 0.33 | -1.36 - -1.18 |  |
| **Cytotoxic lymphocytes** |  |  |  |  |  |  | 0.5 |
| Mean (SD) | 0.98 (0.98) | 0.73 (0.77) | 1.25 (0.93) | 0.84 (0.56) | 0.75 (0.67) | 1.17 (0.90) |  |
| Median (Q1 - Q3) | 0.68 (0.51 - 1.19) | 0.77 (0.16 - 1.23) | 1.12 (0.39 - 1.76) | 0.71 (0.50 - 1.42) | 0.64 (0.37 - 1.40) | 1.41 (1.29 - 1.49) |  |
| Min - Max | -0.79 - 4.00 | -0.59 - 2.32 | 0.14 - 3.30 | 0.17 - 1.42 | -0.43 - 1.78 | -0.59 - 2.02 |  |
| **B lineage** |  |  |  |  |  |  | 0.4 |
| Mean (SD) | 0.67 (0.66) | 0.36 (0.74) | 0.97 (1.34) | 1.08 (0.81) | 0.57 (0.37) | 0.96 (1.19) |  |
| Median (Q1 - Q3) | 0.58 (0.30 - 0.83) | 0.40 (-0.06 - 0.68) | 0.56 (0.09 - 0.98) | 0.80 (0.65 - 1.15) | 0.68 (0.35 - 0.78) | 1.24 (-0.04 - 1.84) |  |
| Min - Max | -0.40 - 2.61 | -0.64 - 2.36 | -0.27 - 3.96 | 0.36 - 2.45 | -0.14 - 1.18 | -0.83 - 2.30 |  |
| **NK cells** |  |  |  |  |  |  | 0.9 |
| Mean (SD) | -0.46 (0.45) | -0.40 (0.51) | -0.05 (1.07) | -0.18 (0.36) | -0.29 (0.63) | -0.30 (0.85) |  |
| Median (Q1 - Q3) | -0.49 (-0.98 - -0.11) | -0.30 (-0.89 - 0.02) | -0.54 (-0.69 - 0.32) | -0.21 (-0.47 - 0.09) | -0.54 (-0.76 - 0.27) | -0.56 (-0.99 - 0.13) |  |
| Min - Max | -1.02 - 0.33 | -1.01 - 0.39 | -1.01 - 2.44 | -0.57 - 0.29 | -1.02 - 0.85 | -1.00 - 1.19 |  |
| **Monocytic lineage** |  |  |  |  |  |  | 0.10 |
| Mean (SD) | 2.30 (0.96) | 2.02 (1.22) | 3.15 (0.99) | 2.97 (1.06) | 2.28 (0.50) | 2.56 (0.94) |  |
| Median (Q1 - Q3) | 1.97 (1.53 - 3.12) | 2.00 (1.11 - 2.96) | 3.19 (2.56 - 3.76) | 2.86 (2.48 - 3.88) | 2.25 (1.87 - 2.73) | 2.65 (1.96 - 3.12) |  |
| Min - Max | 0.48 - 4.06 | -0.03 - 4.16 | 1.60 - 4.98 | 1.53 - 4.13 | 1.47 - 3.05 | 1.13 - 3.86 |  |
| **Myeloid dendritic cells** |  |  |  |  |  |  | 0.4 |
| Mean (SD) | 0.20 (1.36) | -0.38 (0.44) | -0.05 (1.03) | 0.08 (0.74) | -0.26 (0.42) | -0.29 (0.48) |  |
| Median (Q1 - Q3) | -0.16 (-0.43 - 0.22) | -0.39 (-0.81 - 0.10) | -0.51 (-0.63 - 0.25) | 0.19 (-0.60 - 0.65) | -0.37 (-0.57 - 0.18) | -0.24 (-0.61 - 0.13) |  |
| Min - Max | -0.61 - 6.11 | -0.97 - 0.23 | -0.96 - 2.32 | -0.77 - 0.92 | -0.95 - 0.34 | -0.99 - 0.22 |  |
| **Neutrophils** |  |  |  |  |  |  | 0.4 |
| Mean (SD) | 1.26 (0.35) | 0.91 (0.49) | 1.33 (0.64) | 1.14 (0.56) | 1.19 (0.36) | 1.17 (0.44) |  |
| Median (Q1 - Q3) | 1.17 (1.01 - 1.34) | 1.04 (0.69 - 1.24) | 1.19 (0.98 - 1.67) | 0.91 (0.88 - 1.11) | 1.16 (0.94 - 1.53) | 1.17 (0.86 - 1.58) |  |
| Min - Max | 0.81 - 2.19 | -0.16 - 1.50 | 0.67 - 2.91 | 0.70 - 2.11 | 0.55 - 1.74 | 0.52 - 1.70 |  |
| **Endothelial cells** |  |  |  |  |  |  | 0.2 |
| Mean (SD) | 1.59 (0.62) | 0.99 (0.72) | 1.64 (0.88) | 1.70 (0.57) | 1.34 (0.47) | 1.19 (0.67) |  |
| Median (Q1 - Q3) | 1.50 (1.25 - 2.14) | 1.30 (0.38 - 1.49) | 1.49 (0.74 - 1.86) | 1.50 (1.37 - 1.78) | 1.16 (0.98 - 1.68) | 1.23 (0.73 - 1.89) |  |
| Min - Max | 0.18 - 2.91 | -0.29 - 1.77 | 0.70 - 3.30 | 1.22 - 2.65 | 0.87 - 2.37 | 0.19 - 1.89 |  |
| **Fibroblasts** |  |  |  |  |  |  | 0.5 |
| Mean (SD) | 7.19 (1.32) | 7.10 (1.02) | 7.79 (0.89) | 6.93 (1.56) | 7.64 (1.07) | 7.89 (0.57) |  |
| Median (Q1 - Q3) | 7.59 (5.78 - 8.39) | 7.28 (6.27 - 8.07) | 7.98 (7.55 - 8.35) | 7.15 (5.68 - 7.91) | 7.65 (7.25 - 8.50) | 7.86 (7.52 - 7.98) |  |
| Min - Max | 5.37 - 8.92 | 5.24 - 8.41 | 5.53 - 8.87 | 5.06 - 8.85 | 5.19 - 9.07 | 7.23 - 8.91 |  |

Supplementary Table 21: Site of metastasis and immuno markers in the retrospective and ambispective cohorts

| **Characteristic** | **No relapse** | **Relapse (non-liver)** | **Relapse (liver)** | **p-value** |
| --- | --- | --- | --- | --- |
| **IS (Retrospective)** |  |  |  | 0.5 |
| IS0-2 | 94 (85%) | 18 (86%) | 21 (95%) |  |
| IS3 | 17 (15%) | 3 (14%) | 1 (4.5%) |  |
| Unknown | 25 | 3 | 2 |  |
| **MCP classes (Retrospective)** |  |  |  | >0.9 |
| Immune Low | 70 (63%) | 13 (59%) | 14 (70%) |  |
| Stromal | 28 (25%) | 5 (23%) | 4 (20%) |  |
| Immune High | 14 (13%) | 4 (18%) | 2 (10%) |  |
| Unknown | 24 | 2 | 4 |  |
| **ISIC (Retrospective)** |  |  |  | >0.9 |
| Low | 28 (47%) | 4 (57%) | 3 (43%) |  |
| High | 31 (53%) | 3 (43%) | 4 (57%) |  |
| Unknown | 77 | 17 | 17 |  |
| **TuLIS-like (Retrospective)** |  |  |  | 0.3 |
| Low | 79 (71%) | 17 (81%) | 19 (86%) |  |
| High | 32 (29%) | 4 (19%) | 3 (14%) |  |
| Unknown | 25 | 3 | 2 |  |
| **IS (Prospective)** |  |  |  | 0.4 |
| IS0-2 |  | 20 (87%) | 52 (95%) |  |
| IS3 |  | 3 (13%) | 3 (5.5%) |  |
| Unknown |  | 2 | 5 |  |
| **MCP classes (Prospective)** |  |  |  | 0.10 |
| Immune Low |  | 12 (48%) | 32 (53%) |  |
| Stromal |  | 13 (52%) | 21 (35%) |  |
| Immune High |  | 0 (0%) | 7 (12%) |  |
